# CRIPTO’s multifaceted role in driving aggressive prostate cancer unveiled by in vivo, organoid, and patient data

**DOI:** 10.1038/s41388-024-03230-x

**Published:** 2024-11-26

**Authors:** Elisa Rodrigues Sousa, Simone de Brot, Eugenio Zoni, Soheila Zeinali, Andrea Brunello, Mario Scarpa, Marta De Menna, Federico La Manna, Allen Abey Alexander, Irena Klima, David W. Freeman, Brooke L. Gates, Domenico A. Cristaldi, Olivier T. Guenat, Boudewijn P. T. Kruithof, Benjamin T. Spike, Panagiotis Chouvardas, Marianna Kruithof-de Julio

**Affiliations:** 1 Urology Research Laboratory, Department for BioMedical Research, University of Bern, Bern, Switzerland; 2 COMPATH, Institute of Animal Pathology, University of Bern, Bern, Switzerland; 3 Department of Urology, Inselspital, Bern University Hospital, University of Bern, Bern, Switzerland; 4 Organs-on-chip Technologies Laboratory, ARTORG Center for Biomedical Engineering Research, University of Bern, Bern, Switzerland; 5 Department for BioMedical Research, Translational Organoid Research, University of Bern, Bern, Switzerland; 6 Huntsman Cancer Institute, Department of Oncological Sciences, University of Utah, Salt Lake City, USA; 7 CellDynamics Isrl, Bologna, Italy; 8 Department of Cardiology, Leiden University Medical Center, Leiden, The Netherlands; 9 Department of Cell and Chemical Biology, Leiden University Medical Center, Leiden, The Netherlands

**Keywords:** Prostate cancer, Cancer stem cells

## Abstract

CRIPTO (or CR-1 or TDGF1) is a protein that plays an active role in tumor initiation and progression. We have confirmed that increased expression of CRIPTO is associated with clinical and prostate-specific antigen (PSA) progression in human prostate tissues. Our approach involved gaining insight into the role of CRIPTO signaling in castration-resistant *Nkx3.1-*expressing cells (CARNs), targets for oncogenic transformation in prostate cancer (PCa), by integrating the existing *Cripto*^flox/flox^ into CARNs model. The most aggressive stage was modeled using an inducible Cre under control of the *Nkx3.1* promoter conferring *Nkx3.1* inactivation and driving *Pten* inactivation, oncogenic *Kras* activation, and lineage tracing with yellow fluorescence protein (EYFP) upon induction. Our findings provide evidence that selective depletion of *Cripto* in epithelial cells in vivo reduced the invasive phenotype, particularly in more advanced tumor stages. Moreover, in vitro experiments with *Cripto* overexpression demonstrated alterations in the physical features of organoids, which correlated with increased tumorigenic activity. Transcriptomic analyses revealed a unique *CRIPTO/MYC* co-activation signature associated with PSA progression in a human PCa cohort. Taken together, our data highlights a role for CRIPTO in tumor invasiveness and progression, which carries implications for biomarkers and targeted therapies.

## Introduction

CRIPTO (CR-1, TDGF1) is a glycosylphosphatidylinositol (GPI)-anchored and secreted protein founding member of the vertebrate Epidermal Growth Factor-Cripto/FRL-1/Cryptic (EGF-CFC) gene family [1–3]. CRIPTO plays a crucial role in regulating stem cell-associated signaling pathways, promoting cellular plasticity, and maintaining the stem cell state; it has been identified as a significant biomarker to investigate. *CRIPTO* is typically highly expressed in embryonic tissues and decreases during postnatal life under normal conditions; however, in certain pathological conditions, CRIPTO expression may be reactivated [4–10]. Beyond prostate cancer (PCa) [9, 11], CRIPTO has been implicated in colon [8], lung [12], breast [13], liver (hepatocellular carcinoma) [4], and brain (glioblastoma) [14]. Additionally, it has been shown to play a significant role in promoting epithelial-to-mesenchymal transition, which is closely linked to the invasive nature of tumor cells [11]. Still, further investigation into the role of CRIPTO in prostate tissue is necessary to better understand its contribution to PCa pathology.

To effectively study tumor initiation, progression, and response to therapy in PCa, researchers have extensively relied on genetically engineered mouse models (GEMMs) and prostate organoids. These models are crucial as they provide robust systems that mimic various stages of PCa, starting from the early development of prostatic intraepithelial neoplasia (PIN) to the subsequent progression into invasive adenocarcinoma and the formation of metastases.

Distinct stages of disease can be modeled by a series of genetic perturbations in PCa GEMMs including the loss-of-function of *Nkx3.1* to recapitulate early PIN (N) [15–18], additional loss of *Pten* expression to mimic advanced adenocarcinoma (NP) [19], and activation of oncogenic mutant of *K-ras* (G12D) to mimic metastatic disease (NPK) [20]. The R26R-EYFP line was crossed into these various models to allow lineage tracing. Detailed genotypes and newly generated GEMMs that incorporate *Cripto*^flox/flox^ allele [21] (NC, NPC, and NPKC) are indicated in Supplementary Table 1.

Castration-resistant *Nkx3.1*-expressing (CARNs) cells are a specific prostate epithelial luminal stem-like cell subpopulation representing only 0.7% of total epithelial cells in the prostate [22]. These cells are exclusively luminal and serve as a target for oncogenic transformation and the propagation of prostate cancer [22]. Remarkably, studies have revealed that within just 4 weeks, the combined deletion of *Pten* and activation of *Kras* in CARNs leads to the rapid formation of high-grade PIN and carcinoma, with evidence of micro invasion [22]. Interestingly, these tumors express high levels of CRIPTO (unpublished Rodrigues Sousa). We have developed a unique transgenic mouse model with a prostate-specific inducible knockout of *Cripto* in CARNs.

Here, we demonstrate that suppressing *Cripto* in vivo significantly decreases invasive cell behavior in aggressive tumors, highlighting a critical oncogenic function of CRIPTO in advanced PCa. Additionally, our findings suggest that CRIPTO signaling is pivotal in promoting the proliferation and aggressiveness of stem cell-like cells, known for their role in castration-resistant PCa. This condition is closely linked to metastasis, resistance to treatment, and unfavorable patient survival rates. These findings position CRIPTO as a potential biomarker and therapeutic target, enriching our comprehension of PCa evolution and advancement.

## Results

### High CRIPTO expression correlates with clinical and PSA progression in human prostate cancer

**Fig. 1:**
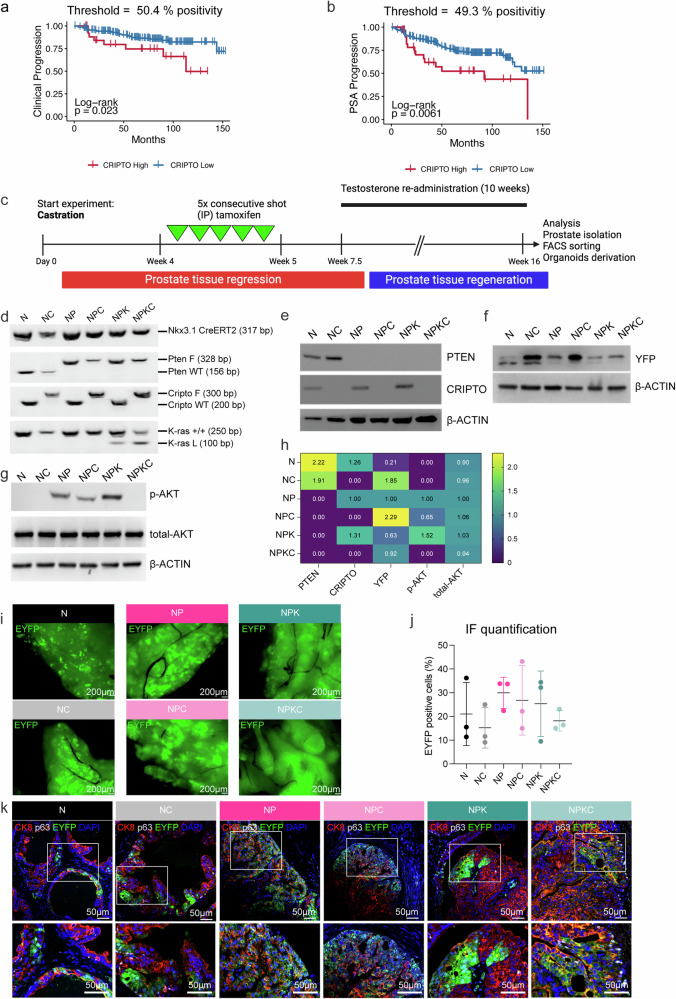
CRIPTO expression in human PCa and establishment of a new mouse model to investigate CRIPTO signaling in PCa progression. Evaluation of CRIPTO expression in a tissue microarray of primary prostate cancer tissues (*n* = 209 patients), part of the European Multicenter High-Risk Prostate Cancer Clinical and Translational Research group (EMPaCT, 540 radical prostatectomies (RP), 52 lymph node metastases). Kaplan-Meyer analyses show the association of high CRIPTO expression with clinical (**a**) and PSA (**b**) progression in EMPaCT cohort (clinical progression: log-rank, *p* = 0.023; HR = 2.4539, (likelihood ratio *p* = 0.04294); PSA progression: log-rank, *p* = 0.0061; HR = 2.2617, (likelihood ratio *p* = 0.01339)). **c** Schematic experimental timeline in castration and regenerated state in N, NC, NP, NPC, NPK, and NPKC genotypes. Male mice 8 weeks of age of the indicated genotypes were castrated and, after 1 month, induced with tamoxifen for 5 consecutive daily injections (i.p.). After 2.5 weeks, mice were subjected to weekly re-administration of testosterone for a total amount of 10 weeks. At the end of the 10 weeks of testosterone re-administration, prostates are isolated and processed for further analysis. Created with BioRender.com. **d** Genomic DNA was isolated from indicated genotypes, and PCR was used to confirm *Nkx3.1/Cripto/Pten/K-ras* (G12D) transgenes. **e**–**g** Western blot analyses using whole-prostate lysates were generated from indicated genotypes to confirm genotypes at a protein level. Representative western blot displaying the levels of PTEN, CRIPTO (**e**), EYFP (**f**), and p(Ser473)/total -AKT (**g**). **h** Representative heatmap of Western Blot analyses quantification. **i** Fluorescent microscopy acquisition was used to validate system induction, displaying strong endogenous EYFP (green) expression in all mice. Scale bar = 200 µm. **j** EYFP-protein levels were quantified by IF staining (two-tailed unpaired *t-*test, *p* > 0.05). **k** Representative IF images (low-power top images, high-power bottom images) to determine the expression of luminal marker CK8 (red) and basal marker p63 (white) in prostates derived from EYFP^+^ (green) cells; all cells were visualized with nuclear stain, DAPI (blue). Scale bar = 50 µm.

To explore the expression pattern of CRIPTO in PCa, we analyzed CRIPTO protein expression on a tissue microarray (TMA) consisting of primary prostate cancer tissues from the European Multicenter High-Risk Prostate Cancer Clinical and Translational research group (EMPaCT). To address intratumor heterogeneity, samples gathered in EMPaCT (Supplementary Table 2) present multiple tumor samples taken from the index lesion, including variably differentiated areas of each tumor (ranging from 1 to 4) (Supplementary Fig. 1.1a) [23]. Furthermore, patients were categorized based on different Gleason scores (ranging from 1 to 5), displaying distinct overall survival rates (Supplementary Fig. 1.1b). Haematoxylin and eosin (H&E) and immunohistochemistry (IHC) stainings were performed, and analyzed cores showed CRIPTO expression was highest in tumor cells, which constituted a greater proportion of total cells compared to stromal cells (Supplementary Fig. 1.1c, d). Among these tumor cells, a high/low percentage of CRIPTO-positive tumor cells were identified (Supplementary Fig. 1.1e–g). Survival analysis of 209 patients demonstrated a positive correlation between high CRIPTO expression and both clinical (Fig. 1a) and PSA progression (Fig. 1b).

Overall, the observed positive association between CRIPTO expression levels and clinical factors, as well as PSA progression, indicates its potential as a prognostic marker forecasting disease severity and patient prognoses.

### Establishment of a new mouse model to investigate CRIPTO signaling in PCa progression

We utilized mice that express an inducible Cre recombinase controlled by the *Nkx3.1* promoter (*Nkx3.1*^CreERT2/CreERT2^). This setup enables precise control over gene recombination in CARN cells through genetic alterations involving *Pten*^flox/flox^, *Cripto*^flox/flox^, and *K-ras*^LSL/+^ (Supplementary Table 1). Furthermore, we activated the *R26R*^EYFP/EYFP^ allele to conduct lineage tracing employing the yellow fluorescent protein (EYFP) to visualize regenerated prostate tissue after castration. The experimental layout used is referred to as the *Castration Setting*. In short, male mice at the age of 8 weeks underwent surgical castration and subsequent tamoxifen induction and transitioned through phases of prostate regression and regeneration (Fig. 1c). Prostate tissues were isolated, genotypes were validated (Fig. 1d, Supplementary Table 3), and the respective protein levels were assessed and quantified through western blot analysis (Fig. 1e–h). Quantitative analysis revealed lower protein levels of pAKT in NPC and NPKC genotypes compared to NP and NPK, respectively, while total AKT levels were unaffected by *Cripto* knockout (Fig. 1g, h). YFP+/- cells were isolated from each genotype by FACS (Supplementary Fig. 1.2a–l). To confirm the ongoing presence of CARNs in the regenerated prostates—cells responsible for generating new tissue after castration and subsequent testosterone re-administration – we utilized both the inherent EYFP fluorescence (Fig. 1i) and quantification of EYFP immunostaining (Fig. 1j). Bipotentiality of the transformed luminal CARNs was confirmed by basal (p63) and luminal cytokeratin 8 (CK8) markers immunostaining (Fig. 1k) [22, 24].

Morphological characterization (Supplementary Fig. 1.3a) and measurements of prostate glands weight (mg) normalized to body weight (g) (Supplementary Fig. 1.3b) correlated with previously reported PCa genotypes, showing that more advanced stages present with a significantly enlarged prostate gland compared to non-aggressive tumors [25]. Our results confirmed that CRIPTO levels in different genotypes do not contribute to significant changes in the prostate weight (Supplementary Fig. 1.3b), while mouse body weight was different only in the N background in the *Castration Setting*. Overall, body weight followed a similar pattern in all genotypes over time, with an average gain of 6–8 g (Supplementary Fig. 1.3c, d).

### CRIPTO deletion in CARNs correlates with a reduction of invasiveness in aggressive lethal PCa

**Fig. 2:**
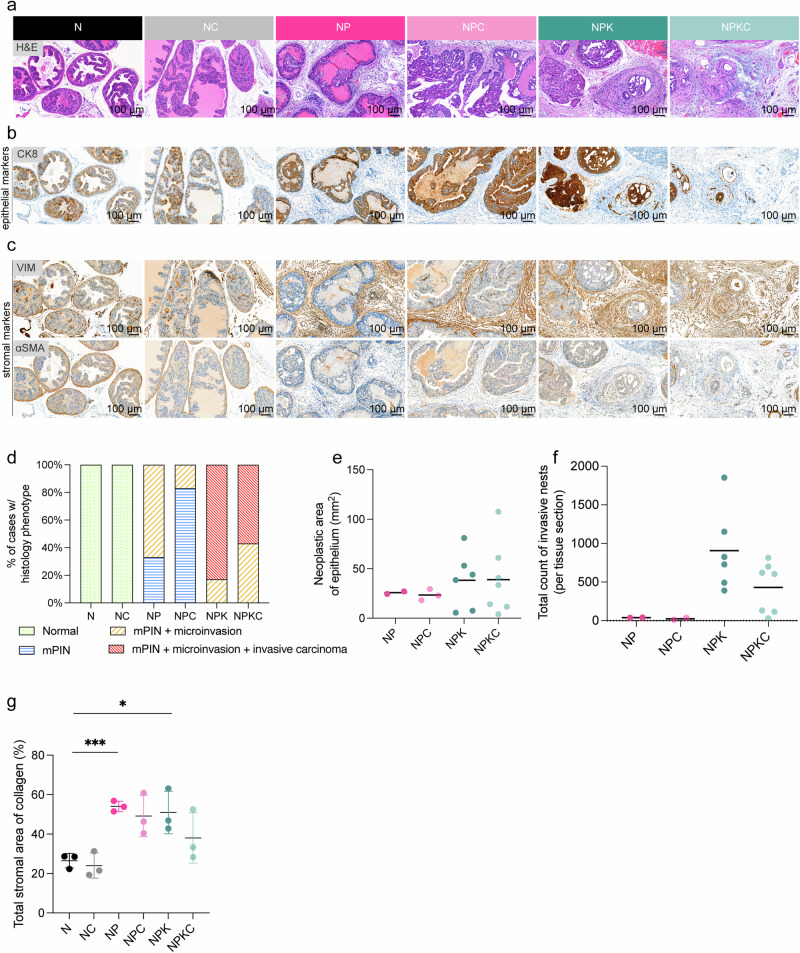
Histologic quantitative and qualitative analysis of neoplastic growth and level of invasion on murine prostate tissue sections. Published N, NP, and NPK models and newly generated NC, NPC, and NPKC prostates were analyzed for microscopic changes to the prostate epithelium and stroma via histopathological evaluation. *Cripto* knockout induces a reduction in invasive carcinoma. **a** Representative haematoxylin and eosin (H&E) staining of prostate tissue in each genotype (N, NC, NP, NPC, NPK, NPKC), shown at high power, demonstrates the development of neoplastic changes consistent with mPIN and invasive adenocarcinoma. Scale bar = 100 µm. Direct serial section unavailable for NPC sample. **b** CK8 (epithelial marker) immunohistochemical (IHC) staining was performed for epithelium characterization and quantitative analysis of neoplastic non-invasive vs. invasive growth. Scale bar = 100 µm. **c** IHC staining for VIMENTIN and αSMA expression was performed for stromal characterization and detection of epithelial-mesenchymal transition (VIMENTIN). Scale bar = 100 µm. **d** Bar plot of the percentage of mice representative of a specific epithelial phenotype (normal (green), mPIN (blue), mPIN + microinvasion (yellow), mPIN + invasive carcinoma (red)) (see Supplementary Table S4.2 for detailed values). **e** Histological scores of CK8 stained prostate tissues representative of the total neoplastic area of the epithelium (mm^2^) (two-tailed unpaired *t-*test, *p* = 0.97). **f** Histological scores of CK8 stained prostate tissues represent the total count of invasive nests per tissue section (NP, NPC, NPK, and NPKC) (NPK vs. NPKC, Mann-Whitney *t-*test, *p* = 0.10). **g** IF analysis of the total stromal area of collagen (%) in all groups (N, NC, NP, NPC, NPK, NPKC) (two-tailed unpaired *t-*test, N vs NP, ***p < 0.0005; N vs. NPK, *p = 0.02; N vs. NC, *p* = 0.59; NP vs. NPC, *p* = 0.47; NPK vs. NPKC, *p* = 0.25).

We assessed alterations in the prostate glandular architecture and histology based on H&E (Fig. 2a, Supplementary Table 4.1) and IHC for epithelial (CK8) (Fig. 2b, Supplementary Table 4.1), epithelial to mesenchymal transition (VIMENTIN) and stromal (alpha-smooth muscle Actin (αSMA)) (Fig. 2c, Supplementary Table 4.1) markers. In our histopathological analysis (Table 1 and Supplementary Table 4.2), consistent with previously published GEMMs [15–17, 25–28], we observed the following: (i) The N group exhibited a benign prostate gland and unremarkable stroma without significant histopathological changes [15, 17, 29]. (ii) The NP group displayed neoplastic prostate epithelium, indicative of murine PIN (mPIN), with few or no remaining profiles of benign glands [19, 26, 27]. (iii) The NPK group was characterized by more extensive mPIN, often with cystic dilation, compared to NP. This group also exhibited variable levels of mPIN microinvasion and invasive carcinoma embedded in a desmoplastic stroma (Fig. 2d, Supplementary Table 4.2). The evaluation of the relative area of neoplastic gland tissue (i.e., % of epithelium with neoplastic transformation) revealed that most cases from all groups with the presence of neoplastic growth showed levels above 95% (Table 1). When comparing the absolute area of the neoplastic epithelium (mPIN plus invasive carcinoma) on the examined tissue section, no difference was evident between NPKC and NPK (Fig. 2e). When comparing the absolute and relative (i.e., % of neoplastic tissue, which is invasive) levels of tumor invasive growth instead, a marked variation within group NPK, together with overall lower levels in NPKC compared to NPK were observed. This trend is supported by the individual invasive neoplastic nests (Fig. 2f). Interestingly, collagen type I quantification by IF staining significantly correlates with tumor progression (Fig. 2g, Supplementary Fig. 2a–c, Supplementary Table 4.3). Comparing the controls (N, NP, and NPK) and experimental groups (NC, NPC, and NPKC), we observed a trend with the mean values (%) of stromal area positive for collagen type I decreasing by 12.9% in the most aggressive stage NPKC, compared to NPK (mean value NPK = 50.93%, mean value NPKC = 38.03%).

**Table 1:** Immunohistochemical analyses of mouse prostate tumors extent and level of invasion.

Group	Area glandular epithelium (mm^2^)						Count of invasive nests
	Total^a^	Benign	Neoplastic^b^	%	Invasive	%	
N	4	4	0	0	0	0	NA
NC	6.7	6.7	0	0	0	0	NA
NP	25.6	1	24.6	96.1	0.01	0	40
NP	28.1	1	27	96.3	0.01	0	37
NPC	29.4	0	29.4	100	0.01	0	12
NPC	21.4	3.3	18.2	84.8	0.02	0.1	37
NPK	5.5	0	5.5	100	0.3	5.4	825
NPK	7.8	0.3	7.5	96.2	1.59	21.2	1153
NPK	44	0	44	100	0.15	0.3	492
NPK	41.4	2.8	38.5	93.2	0.18	0.5	391
NPK	81.1	0	81.1	100	0.4	0.5	1852
NPK	53.1	0	53.1	100	0.31	0.6	729
NPKC	4^c^	0	4^c^	100	0.05	1.2	132
NPKC	40.7	0	40.7	100	0.19	0.5	604
NPKC	14	0	14	100	0.02	0.1	30
NPKC	12.5	1	11.5	91.9	0.11	1	115
NPKC	33.6	0	33.6	100	0.5	1.5	701
NPKC	61	0	61	100	0.25	0.4	620
NPKC	110	2.3	107.6	97.9	0.32	0.3	814

Quantitative analysis of CK8 staining in *Castration Setting*.

*NA* not applicable.

^a^Benign and neoplastic; CK8 positive.

^b^mPIN and invasive combined.

^c^Incomplete prostatic section was available for analysis.

In summary, the loss of CRIPTO influenced the progress of advanced prostate cancer stages, leading to a decrease in invasive carcinoma and changes in stromal composition.

### Generation of mouse prostate organoids

**Fig. 3:**
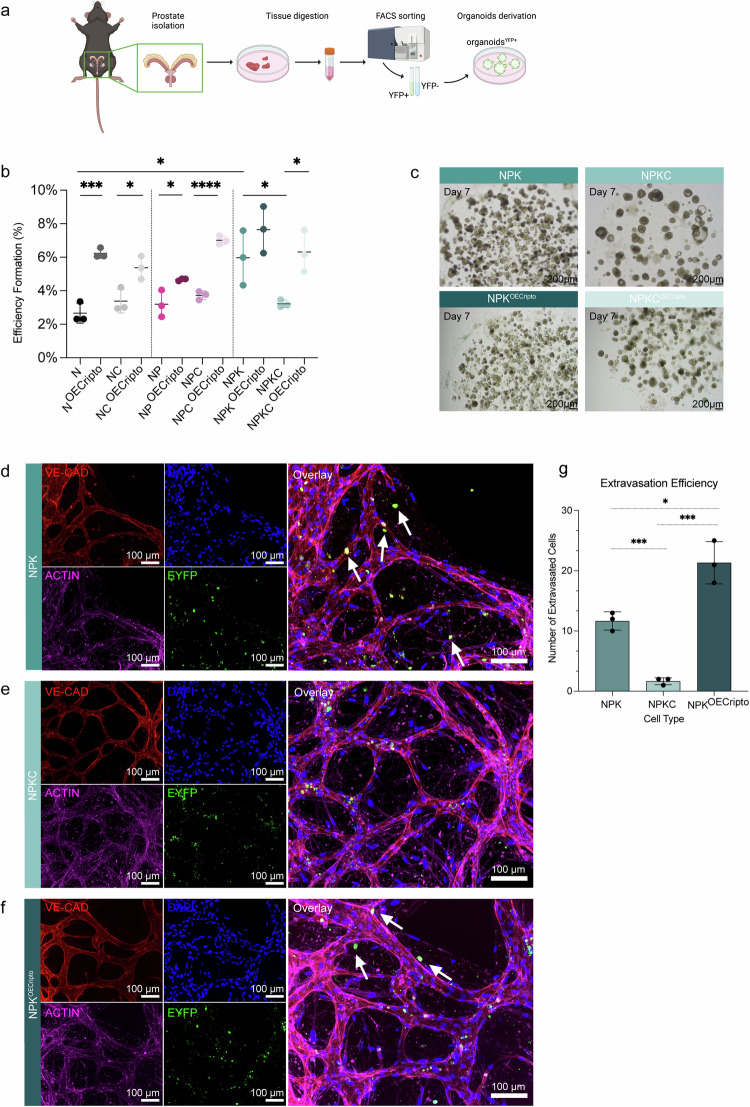
Establishment of mouse prostate organoids and organoids formation efficiency. Tumor cell extravasation correlates with metastatic potential in vitro*.* Mouse prostate organoids were derived and used as an efficient in vitro model to investigate the oncogenic role of CRIPTO. **a** Schematic of experimental protocol for mouse prostate organoids derivation and culture. Created with BioRender.com. **b** Efficiency formation calculations per Matrigel® dome (*n* = 3) in indicated genotypes (two-tail unpaired *t-*test, N vs. N^OECripto^, ***p = 0.0008; NC vs. NC^OECripto^, *p = 0.025; NP vs. NP^OECripto^, *p = 0.034; NPC vs. NPC^OECripto^, ****p = 0.0001; NPK vs. NPKC, *p = 0.04; NPKC vs. NPKC^OECripto^, *p = 0.013; N vs. NPK, *p = 0.03). **c** Representative brightfield pictures specifically for NPK background (NPK, NPKC, NPK^OECripto^, NPKC^OECripto^). Scale bar = 200 µm. **d**–**f** Confocal projection of a representative region of the microvasculature-on-chip stained for different markers (VE-CADHERIN (red), ACTIN (magenta), EYFP (green), DAPI (blue)) in NPK (**d**), NPKC (**e**), and NPK^OECripto^ (**f**). White arrows show the extravasated cells located outside the microvessels within hydrogel matrix. Scale bar = 100 µm. **g** Extravasation efficiency calculations per chip (*n* = 3) in indicated genotypes (two-tail unpaired *t*-test, NPK vs. NPK^OECripto^, *p = 0.012; NPK vs. NPKC, ***p = 0.0004; NPKC vs NPK^OEcripto^, ***p = 0.0007).

To explore the potential role of CRIPTO in modulating tumorigenic characteristics in CARNs, we cultured organoids from our models (Fig. 3a). In brief, we collected tissue from regenerated prostates, isolated the EYFP+ population via flow cytometry (Supplementary Fig. 1.2a–l), and then cultured these single cells in Matrigel® (Fig. 3a). Subsequently, we conducted lentiviral infection to induce the overexpression of *Cripto* in the organoids (Supplementary Fig. 3.1a–c).

IF stainings confirmed that most organoids were uniformly composed of EYFP+ cells (Supplementary Fig. 3.2a, c, e). Furthermore, organoids present a distinct organization: an outer layer of cells expressing the basal marker p63, inner cells positive for the luminal marker CK8, and a few ‘intermediate’ cells co-expressing both luminal and basal markers, thus forming complex multilayered structures that resembled phenotypes seen in living organisms (Supplementary Fig. 3.2b, d, f). Successful generation of *Cripto* overexpression was confirmed at mRNA levels in all three backgrounds (Supplementary Fig. 3.3a–c).

### CRIPTO overexpression in organoids promotes their proliferation and efficiency formation in vitro

To determine relative organoid-forming efficiency, we counted the number of organoids formed per Matrigel® dome (*n* = 3) after 7 days of culture normalized to the initial number of single cells seeded (Fig. 3b, c). Our results showed that the organoid-forming efficiency significantly correlates with tumor progression (N vs. NPK, *p = 0.03) and CRIPTO expression (Fig. 3b and Supplementary Table 5). Of note, *Cripto* overexpression, particularly in the less aggressive stages of the disease (N vs. N^OECripto^, ***p = 0.0008), significantly increased organoid-forming efficiency. The higher efficiency of NPK (compared to N and NP) is dependent on CRIPTO expression as efficiency is reduced in NPKC relative to NPK (NPK vs. NPKC, *p = 0.04 (Fig. 3b, c)), and this can be rescued by overexpression of *Cripto* as detected in NPKC^OECripto^ (Fig. 3b, c).

N EYFP+ cells in organoids (Supplementary Fig. 3.3d) showed a linear growth over 7 days of culture as assessed by CellTiter-Glo® 3D (Supplementary Figure 3.3e). In contrast, the NP background (Supplementary Fig. 3.3f) presented an initial exponential growth (day 5) followed by a plateau phase from day 5 to day 7 (Supplementary Fig. 3.3g). In the NPK background (Supplementary Fig. 3.3h), NPK and NPK^OECripto^ exhibited the fastest growth rate, with exponential growth within 4 days, followed by a plateau phase. On the contrary, NPKC and NPKC^OECripto^ organoids needed an additional 24 h to reach the plateau phase (Supplementary Fig. 3.3i). Histologically, organoids derived from OECripto conditions consistently present with smaller diameters and absent (or neglectable) central necrosis, confirming a higher proliferative phenotype.

These results demonstrated that the derivation of murine PCa organoids may serve as an efficient in vitro platform for gene editing in a self-renewing multilayered epithelium. Overall, CRIPTO stimulated proliferation and organoids forming efficiency indicating its oncogenic potential.

### CRIPTO expression plays a role in the extravasation potential of single cells from organoids in a microvasculature-on-chip system

To further validate the oncogenic potential of CRIPTO, we tested the extravasation potential of EYFP+ cells from organoids in vitro by using a microvasculature-on-chip system (previously reported) to detect extravasation potential in a self-assembled microvasculature network (Supplementary Fig. 3.4a–c) [30, 31]. EYFP+ CARNs cells from the NPK, NPKC NPK^OECripto^, were used to determine their potential to extravasate through the endothelial barrier. We show that NPK (Fig. 3d) and NPK^OECripto^ (Fig. 3f) cells exhibited a significantly higher extravasation potential compared to NPKC cells (Fig. 3e) at 24 h post-injection (Fig. 3g). Moreover, it is particularly intriguing to note that the extravasation potential of NPK^OECripto^ cells significantly surpasses the potential of the NPK cells (*p < 0.012*)*. Live imaging showed no significant differences in extravasation behavior over 24 h, but confocal imaging post-fixation and immunostaining demonstrated the presence of NPK^OECripto^ cells surrounding the microvasculature network within the hydrogel matrix (Supplementary Fig. 3.4d–f). This outcome may be attributed to regional microvasculature loss upon cell injection, particularly in NPK^OECripto^ cells, as opposed to NPK.

Interestingly, conditioned media from organoids’ mouse medium across all three conditions (Supplementary Fig. 3.4g–i) altered microvasculature morphology (circled areas in Supplementary Fig. 3.4g–i). This suggests that factors released from the organoids into the medium could impact microvasculature structure. Notably, the effect was more pronounced when vessels were treated with conditioned media from NPK^OECripto^ organoids (Supplementary Fig. 3.4i) and less apparent when endothelial cells were exposed to NPKC organoid-conditioned medium (Supplementary Fig. 3.4h). These results are consistent with previous findings from other experiments using conditioned media, further supporting the reliability and reproducibility of this approach (Supplementary Fig. 3.5).

CRIPTO’s potent influence on extravasation dynamics further underscores its correlation with tumor cell aggressiveness (Fig. 3d–g, and Supplementary Fig. 3.4d–f).

### Organoids’ biophysical and morphological features correlate with CRIPTO expression

**Fig. 4:**
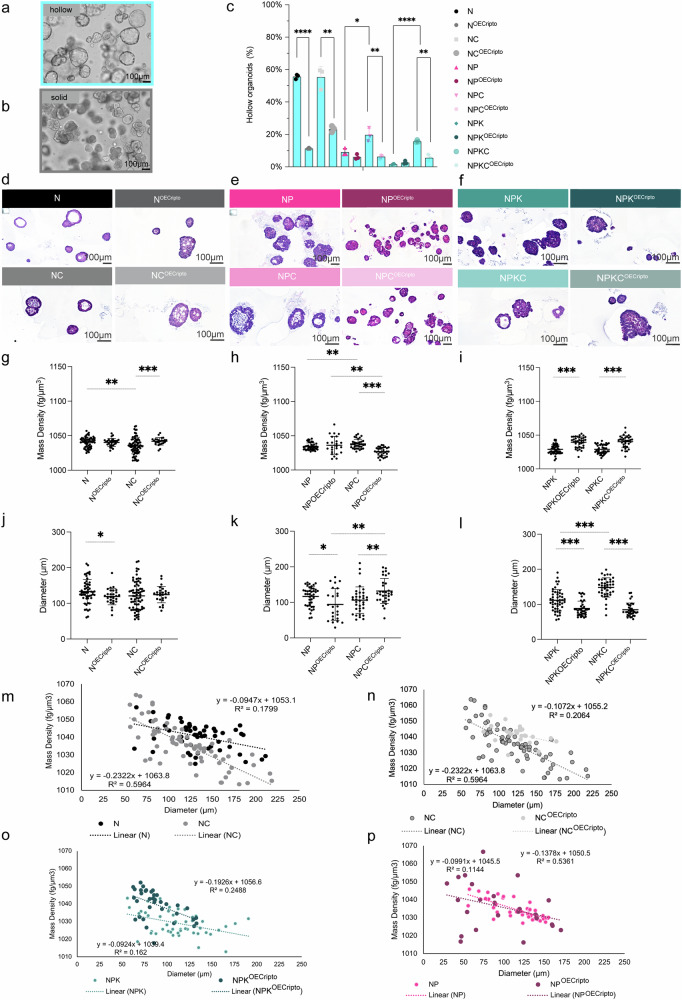
Physical parameter of mouse prostate organoids. The W8 system was employed to perform a detailed physical characterization on all organoids. Representative brightfield images of mouse prostate organoids with hollow (**a**) and solid (**b**) morphology. Scale bar = 100 µm. **c** % of hollow mouse prostate organoids morphology over the total analyzed Matrigel® domes (*n*_domes _= 3) per genotype (two-tailed, unpaired *t-*test, N vs. N^OECripto^, ****p < 0.0001; NC vs. NC^OECripto^, **p < 0.001; NP vs. NPC, *p = 0.017, NPC vs NPC^OECripto^, **p = 0.005; NPK vs. NPKC, ****p < 0.0001; NPKC vs. NPKC^OECripto^, **p = 0.0013. Representative H&E staining of mouse prostate organoids’ sections in the three different background genotypes (N (**d**), NP (**e**), and NPK (**f**)) Scale bar = 100 µm. Physical parameters were assessed with W8 system (two-sample *t*-test, *p < 0.05, **p < 0.01, ***p < 0.001) which allows measurements of mass density (fg/µm^3^) (**g**–**i**), diameter (µm) (**j**–**l**) in all genotypes (*n*_N _= 54, *n*_NOECripto_ = 27, *n*_NC_ = 71, *n*_NCOECripto_ = 25, *n*_NP_ = 41, *n*_NPOECripto_ = 24, *n*_NPC_ = 42, *n*_NPCOECripto_ = 32, *n*_NPK_ = 48, n_NPKOECripto_ = 36, *n*_NPKC_ = 41, *n*_NPKCOECripto_ = 34). **m**–**p** Representative cases of multiple variables plots of mass density and diameter. **m** N (*R*^2^ = 0.1799, slope values in *N* = −0.0947) vs. NC (R^2^ = 0.5964, slope value = −0.2322). **n** NC (*R*^2^ = 0.5964, slope value = −0.2322) vs. NC^OECripto^ (*R*^2^ = 0.2064, slope value = −0.1072). **o** NPK (*R*^2^ = 0.162, slope value = −0.0924) vs. NPK^OECripto^ (*R*^2^ = 0.2488, slope value = −0.1926) (**p**) NP (*R*^2^ = 0.5361, slope value = −0.1378) vs. NP^OECripto^ (*R*^2^ = 0.1144, slope value = −0.0991).

We characterized the biophysical properties of all organoid conditions and observed two morphological patterns: hollow (Fig. 4a) organoids presenting a luminal space and solid (Fig. 4b) aggregations of cells without a luminal space.

Hollow organoids were detected in non-aggressive genotypes (N background), while organoids derived from aggressive stages exhibited solid morphologies (NP and NPK backgrounds) consistent with oncogenic transformation [17, 32]. Notably, *Cripto* overexpression significantly reduced the number of hollow structures, with the most robust difference observed in the N background (N vs. N^OECripto^, ****p < 0.0001) (Fig. 4c). Additionally, in NPK background organoids, a clear reduction of solid structures was detected when *Cripto* was deleted (NPK vs. NPKC, ****p < 0.0001) (Supplementary Fig. 4a). Our results were confirmed via H&E staining semi-qualitative analysis (data not showed) (Fig. 4d–f) and biophysical characterization using the W8 system (CellDynamics iSRL). The W8 system consists of the simultaneous flow-based measurements of organoids’ mass density (fg/µm^3^) (Fig. 4g–i), size (µm) (Fig. 4j–l), and weight (ng) (Supplementary Fig. 4b–d) allowing a robust phenotypical characterization of the organoids’ structures (Fig. 4g–p). Our results confirmed the effects of CRIPTO overexpression on organoids’ mass density, demonstrating an impact on the cross-sectional structure. Specifically, significant differences in mass density were reported in N background (N vs. NC, **p < 0.05; NC vs NC^OECripto^, ***p < 0.001) (Fig. 4g). Several considerations on the biophysical heterogeneity of organoids, both intra-, and inter-population, can be derived from the mass density/size distributions (Fig. 4m–p). Specifically, differences between N and NC trends can be described in both aspects. Their biophysical heterogeneity intra-population appears different in terms of data dispersion (*R*^2^ from linear regression of 0.1799 and 0.5964 for N and NC, respectively) and slope values (−0.0947 and −0.2322 for N and NC, respectively) (Fig. 4m). When comparing their inter-population diversities even though N and NC organoids appear indistinguishable up to the 100 µm size, mass density values start diverging with the further increase of their size, becoming progressively more pronounced the larger the organoids (Fig. 4m). Based on these observations, for organoids larger than 125 µm, the mass density value of 1040 fg/µm^3^, could be defined as a solely biophysical threshold to separate N organoids from a N/NC mixed population thanks to the sampling function of the W8 system. Interestingly, similar trends are also obtained from the biophysical analysis comparison for NC vs NC^OECripto^, with the latter being highest in compaction for organoids larger than 125 µm (Fig. 4n). Also, NPK^OECripto^ presents differences, with an inverse trend, when compared to their controls NPK (***p < 0.001) (Fig. 4i, l, o). From the inter-population mass density/size distribution, potential distinctions can be performed in the 50–100 µm size range, in which NPK^OECripto^ organoids are uniquely present for mass density values higher than 1045 fg/µm^3^. On the other hand, the NP vs NP^OECripto^ comparison highlights an interesting aspect. Although inter-population follows a similar trend, with no evident statistical differences, a notable intra-population biophysical heterogeneity is evident, with a greater mass density dispersion for NP^OECripto^ (*R*^2^ = 0.1144) when compared to the most biophysically uniform NP (*R*^2^ = 0.5361) (Fig. 4p).

Overall, these findings underscore the complex interplay of biophysical factors in organoid development and highlight the importance of considering both intra- and inter-population variability in biophysical analyses.

### A CRIPTO and c-MYC co-activation signature correlates with PSA progression

**Fig. 5:**
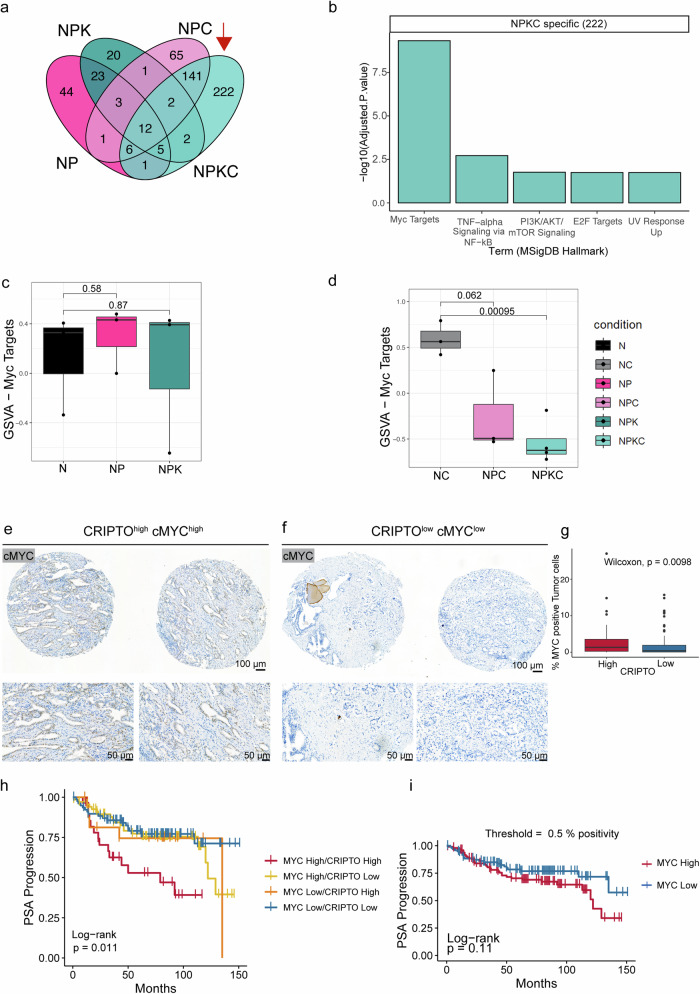
A CRIPTO and cMYC co-activation signature correlates with PSA progression. All data were analyzed from organoids from different genotypes in *Castration Setting*. **a** Venn diagram of differentially expressed genes in the different conditions (N and NC used as control). **b** MsigDB Hallmarks enriched in the NPKC specific differentially expressed genes (Fisher’s exact test, adjusted *p*-values with Benjamini-Hochberg method). **c**, **d** MYC targets signature scoring across all organoid samples using Gene Set Variation Analysis (two-tailed unpaired *t*-test, N vs. NP, *p* = 0.58; N vs. NPK, *p* = 0.87; NC vs. NPC, *p* = 0.062; NC vs. NPKC, ***p = 0.00095). Representative IHC staining for cMYC in CRIPTO^high^cMYC^high^ (**e**) and CRIPTO^low^cMYC^low^ groups (**f**). Scale bar = 100 µm (top), 50 µm (bottom). **g** c-MYC IHC staining quantification in CRIPTO^high^ and CRIPTO^low^ groups (Wilcoxon test, MYC^high^ vs. MYC^low^, ***p = 0.0098)**. h** CRIPTO^high^MYC^high^ expression with PSA progression (log-rank test, *p = 0.011). **i** Kaplan-Meyer analyses showing the association of only MYC^high^ expression with PSA progression (log-rank test, *p* = 0.11; HR = 1.5482, (likelihood ratio *p* = 0.11)).

We performed transcriptomics in organoids derived from the six GEMMs. We conducted differential expression analysis using the N and NC genotypes as control. Comparing the differentially expressed genes, we identified 222 genes specifically differentially expressed in the most aggressive genotype when *Cripto* is KO (NPKC vs NC) (Fig. 5a). These genes show a strong enrichment for oncogenic and proliferation hallmarks, e.g., MYC targets, TNFα signaling via NF-kB, PI3K/AKT/mTOR signaling (Fig. 5b). Subsequently, we focused on MYC targets, the most enriched hallmark, to estimate a signature score using the respective genes. While in control conditions (N, NP, NPK), no significant changes were detected in the expression of cMYC targets (Fig. 5c), CRIPTO knockout correlates with decreased cMYC targets expression and tumor progression (Fig. 5d, Supplementary Fig. 5a).

cMYC protein level quantification was assessed on IHC staining (Supplementary Fig. 5b, c). While not reaching statistical significance, a slight increase in the mean % of cMYC positive cells was observed according to the state of tumor progression and was decreased in the absence of CRIPTO. Interestingly, in both N and NPK backgrounds, reduced mean values of positive cells (%) were observed in NC and NPKC, respectively (Supplementary Fig. 5c). This correlation might indicate a potential regulatory relationship between these two factors. Furthermore, in the context of tumor progression, it suggests a potential link to the development of the behavior of tumors.

We extended our investigation in MYC-CRIPTO interplay, by quantifying cMYC levels in the EMPaCT TMA cohort described in Fig. 1 and Supplementary Fig. 1.1. The staining pattern was predominantly nuclear, with varying degrees of intensity observed among different samples which were stratified in CRIPTO^high^MYC^high^ and CRIPTO^low^MYC^low^ (as depicted by the two representative cases in Fig. 5e, f). No significant staining was observed in the surrounding stromal tissue. Consistent with the known upregulation of MYC in human prostate cancer [33, 34], the IHC staining quantification showed robust MYC expression which correlated with CRIPTO expression (**p = 0.0098) (Fig. 5g). Notably, Kaplan-Meyer survival curve showed a correlation with PSA progression only in the CRIPTO^high^MYC^high^ group (**p = 0.011) (Fig. 5h), while no significant differences were identified in the MYC^high^ only group (*p* = 0.11) (Fig. 5i).

These data suggest a previously unappreciated connection between CRIPTO expression and expression of the MYC proto-oncogene in early and progressing PCa.

## Discussion

The role of CRIPTO signaling in tumor initiation and progression of tumors has been the subject of extensive exploration across various types of cancers. Despite the compelling indications of its potential utility as a clinical biomarker, its practical application in this context remains speculative. Our study aimed to address the significant impact of CRIPTO signaling within diverse PCa models to reaffirm and establish its pivotal role in driving the progression of PCa tumors.

In addition to the significance of Gleason scores in stratifying patients based on overall survival rates, our analyses on CRIPTO expression emphasize the importance of incorporating molecular markers like CRIPTO alongside traditional parameters for more accurate patient risk assessment and personalized treatment strategies in PCa management.

Leveraging newly generated GEMM models, our results indicated that groups with CRIPTO knockout (“C”) exhibited a tendency towards a less aggressive tumor behavior. Specifically, the extent of invasive tumor tissue and the number of invasive tumor clusters were generally lower in groups NPC and NPKC compared to NP and NPK, respectively.

Multiple studies have provided evidence supporting the contribution of the microenvironment to tumorigenesis and progression [35, 36]. Our study indicates that CRIPTO also influences the tumor microenvironment, particularly affecting invasiveness levels and leukocyte infiltration (data not shown) within the tumor stroma.

Our in vitro analysis reveals that organoids exhibit both basal and luminal epithelial layers, along with sustained EYFP expression, indicating their origin from CARNs. Our results show that murine PCa organoids recapitulate in vivo phenotypes known from PCa GEMMs. The use of this powerful in vitro model has the potential to greatly simplify functional genetic studies. By modulating CRIPTO levels in vitro, we investigated the ability of CRIPTO to influence growth, invasiveness, and response to treatments within the isolated epithelium. Prior research into CRIPTO’s role in tumorigenesis has primarily focused on its metastatic potential [9]. While our results on the microvasculature-on-chip model are consistent with these results in terms of extravasation potential, we also showed that increased CRIPTO expression already affects the early stages of the disease, as evidenced by the organoids-forming efficiency quantification and the conditioned media experiments.

There is a notable variance in the physical appearance of organoids depending on the aggressiveness of the model and CRIPTO status, with hollow structures associated with less aggressive phenotypes and solid structures associated with advanced tumors. In comparison to the N and NP backgrounds, NPK organoids also exhibit a heightened proliferation rate.

Intriguingly, our findings validate the influence of CRIPTO overexpression on the mass density of organoids, revealing a discernible effect on the cross-sectional architecture. Cell density [37], the influence of structural compaction [38], and mass density assessments are increasingly recognized as useful in elucidating structural diversity and aggressiveness in 3D cell culture [39].

While previous cross-species analyses of mouse PCa bone metastasis and human PCa identified a co-activation of MYC and RAS pathways as key drivers in metastasis formation (gene signature META-16 [40]), here we focused on primary tumor initiation and progression and showed that MYC targets correlate with CRIPTO expression. This was particularly intriguing as it was associated with PSA progression, further suggesting a potential synergistic role between CRIPTO, MYC targets, and the advancement of prostate cancer-promoting tumorigenesis. Dysregulation of MYC results in the reprogramming of signaling pathways in cancer cells, providing them with selective advantages. MYC deregulation is pervasive, occurring in over 70% of human cancers and correlating with poor prognosis, making the hyperactivated MYC oncoprotein an appealing yet challenging target for drug development due to its “undruggable” characteristics. Despite persistent endeavors and extensive research on targeting MYC, notable progress continues to be made in this field.

In our study, the MYC-CRIPTO interplay is an indirect observation linked to the fact that CRIPTO modulation reduces MYC targets due to CRIPTO deletion. To gain a comprehensive understanding of the mechanistic interplay between these pathways, further investigations are imperative. These studies will delve into elucidating the intricate relationship between MYC and CRIPTO and ascertaining the feasibility of modulating one pathway by inhibiting the other.

## Methods

### EMPaCT

The dataset includes TMAs from 210 primary prostate cancer tissues, provided through EMPaCT and the Institute of Tissue Pathology in Bern. All analyses in this study were performed in accordance with the guidelines of the World Medical Association Declaration of Helsinki 1964, updated in October 2013. All participants provided informed consent, and the study was approved by the Ethical Committees of Bern (CEC ID2015-00128).

Annotations and quantification of positive staining on TMA cores were conducted by QuPath software version v0.0.3 [41], and two datasets per image were exported: TMA measurement and detection measurement. The Kaplan-Meyer curves that assess the correlation between CRIPTO high and low positive cells and disease progression were generated using the R packages *survminer* (V0.4.0) and *survival* (v3.4–0). The optimal cutpoint for CRIPTO high/low stratification was estimated using the maximally selected rank statistic. The optimal threshold for defining high CRIPTO was estimated to be 50.4% (positivity) for clinical progression and 49.3% (positivity) for PSA progression. Metastasis or local recurrence were the events used to define clinical progression. Hazard ratios were estimated by fitting a Cox proportional hazards regression model and their significance was calculated by performing a likelihood ratio test. Data visualizations were generated using the *ggplot2* (v3.4.0), ggpubr (v0.4.0), and *pals* (v1.7) R packages. Data analyses were conducted using R version 4.1.2 and rStudio version 2023.06.1, build 524.

### Genetically engineered mouse models of prostate cancer progression

Unless stated otherwise, animal protocols were performed in C57BL/6 mice at 8–24 weeks of age. They were approved by the Ethical Committee for Animal Experimentation and the Veterinary Authorities of the Canton of Bern (Cantonal Ethical approval BE35/20 and BE102/23). Food and water were administrated *ad libitum*, and mice were housed in individually ventilated cages under a 12-h day/night cycle. Studies were performed using littermate genotypes before surgical castration, which was performed in 8-week-old male mice by bilateral removal of the testis and epididymis, as described by previous protocol [42]. Mice were allowed to regress for 1 month and then induced once daily with five consecutive intraperitoneal (i.p.) injections of 225 mg kg^−1^ tamoxifen (Sigma-Aldrich, T5648) resuspended in corn oil to allow lineage tracing. 2.5 weeks after, mice were re-administered for a period of 10 weeks with weekly subcutaneous (s.c.) injections of testosterone propionate (DHT; 66 mg/kg). At the end of week 10, blood samples were intracardially collected, mice were euthanized, and prostate was collected. EYFP-positive prostates were visualized by ex vivo fluorescence using a CKX53 microscope (Olympus, microscope digital camera DP22) before proceeding with further analysis.

The following transgenic lines, which model the sequential stages of PCa progression, were used. The N (*Nkx3.1*^CreERT2/CreERT2^; *R26R*^EYFP/EYFP^), NP (*Nkx3.1*^CreERT2/CreERT2^; Pten^flox/flox^, *R26R*^EYFP/EYFP^), and NPK (*Nkx3.1*^CreERT2/CreERT2^; *Pten*^flox/flox^; *K-ras*^LSL/+^; *R26R*^EYFP/EYFP^) previously published GEMMs were crossed with Cripto conditional knock out (*Cripto*^flox/flox^) mice to generate NC (*Nkx3.1*^CreERT2/CreERT2^; *Cripto*^flox/flox^; *R26R*^EYFP/EYFP^), NPC (*Nkx3.1*^CreERT2/CreERT2^; *Pten*^flox/flox^; *Cripto*^flox/flox^; *R26R*^EYFP/EYFP^) and NPKC (*Nkx3.1*^CreERT2/CreERT2^; *Pten*^flox/flox^; *K-ras*^LSL/+^; *Cripto*^flox/flox^; *R26R*^EYFP/EYFP^), respectively.

### Mouse prostate organoids derivation

Mouse prostate organoids were generated from prostates isolated from adult N, NC, NP, NPC, NPK, and NPKC males subjected to *Castration Setting* (as previously described). Prostate tissue was collected in Basis medium (Advanced DMEM F12 Serum Free medium (ThermoFisher, 12634028), 2 mM GlutaMAX (ThermoFisher, 35050061), 10 mM HEPES (ThermoFisher, 15630056) and 100 μg/ml Primocin (Invivogen, ant-pm-1)). After mechanical disruption, a washing step in Basis medium (240 g, 5 min) was performed. Enzymatic digestion started by tissue incubation in the enzymatic mix (Basis medium supplemented with 5 mg/ml collagenase type II (ThermoFisher 17101015), 15 μg/ml dNase I (Roche, 10104159001) and 10 μM Y-27632-HCl Rock inhibitor (Selleckchem S1049)). Tissue was incubated at 37 °C for 1–2 h, mixing every 20 min. After a second washing step (300 g, 5 min), digested tissue was passed through a 100-μm cell strainer (VWR, 732,2759) attached to a 50 ml Falcon tube. The cell pellet was incubated in 5 ml cold EC lysis buffer (150 mM NH_4_Cl, 10 mM KHCO_3_, 0.1 mM EDTA) for 10 min at room temperature, followed by a third washing step and centrifugation (240 g, 5 min). The cell pellet was resuspended in 2 ml TrypLE Express (ThermoFisher, 12605028), according to the sample size, and incubated at 37 °C for 15-20 min by mixing every 5 min. The cell pellet was passed through a 70-μm cell strainer (VWR, 732-2758) to further promote single-cell suspension. Viability by trypan blue exclusion was assessed, and single cells were counted and resuspended in the FACS-sorting medium (50% FBS; 50% Basis medium) at a cell density of 5-10*10^6^ cells/ml. After FACS-sorting, EYFP+/- subpopulations were retrieved and embedded in growth factor reduced Matrigel® (Corning, 356231) and plated as 10 μl domes (6,000-8,0000 cells/dome) in a 6-well cell culture plate (Sarstedt, 833.920). The plate was tilted and incubated for 15 min at 37 °C with 5% CO_2_ to favor Matrigel® solidification. Domes were then covered with 1.2–1.5 ml organoids mouse medium which consists of Basis medium supplemented with 1x B27 (ThermoFisher, 17504044), 500 ng/ml R-spondin (Peprotech, 120-38), 1.25 mM N-acetyl-cysteine (Sigma, A9165), 100 ng/ml Noggin (Peproptech, 25038), 500 nM A83-01 (Tocris, 2939), 1 nM Dihydrotestosterone (Fluka Chemica, 10300), 25 ng/ml epidermal growth factor (EGF; Peprotech, AF-100-15), and 10 μM Y-27632-HCl Rock inhibitor. Organoids were top-up on day 3, and medium changed on day 5. Organoids were passed once a week by recovering cells using Dispase II (Sigma, D4693-1G) and TrypLE Express to retrieve single-cell suspension before replating/reseeding. For freezing, organoids were collected 7 days after passaging and dissociated to single cells, resuspended in freezing medium (50% fetal calf serum, 40% basis medium, 10% dimethyl sulfoxide (DMSO) (Sigma, D2650)) and long-term stored at −80 °C or in liquid nitrogen.

### Mouse prostate organoid transduction

Transduction was used to stably integrate mouse teratocarcinoma-derived growth factor 1 (Tdgf1 Myc-DDK-tagged). To perform high-efficiency transductions, organoids were grown until day 7 post passaging, after which cells were trypsinized into single cells. 50,000 cells were used for each condition and mixed with 60 μl concentrated virus and 0.6 μl Polybrene (4 μg/ml) in a 96-well ultra-low attachment plate (ULA). Cells were concentrated at the bottom of the well by a centrifugation step of 600  g for 1 h. After 4–6 h of incubation (37 °C, 5% CO_2_), cells were subsequently re-seeded in Matrigel® domes as described above (Supplementary Fig. 3.1a). Organoids were cultured in organoids mouse medium and incubated at 37 °C (5% CO_2_). Cells were plated in 10 μl Matrigel® domes. Puromycin titration was performed on a single control genotype (NPC) to determine the correct puromycin concentration for the selection (Supplementary Fig. 3.1b), and the optimal puromycin concentration selected was 2 μg/ml (*p = 0.0106; ****p < 0.0001) (Supplementary Fig. 3.1c). Puromycin (2 μg/ml) selection was then applied at day 3 to ensure the culture of infected organoids. Seven days post-infection, organoids were passaged, one part was collected for histopathological analysis (as previously described) (Supplementary Fig. 3.2), and the other was used to isolate RNA to perform molecular analyses.

### Immunofluorescence (IF)

FFPE sections (4 μm) were deparaffinized and rehydrated using xylene and ethanol. Antigen retrieval was performed using a citrate-based buffer (pH 6) (Adipogen, H-3300) or 10 mM Tris (pH=9)/1 mM EDTA/0.05% Tween buffer. Slides were blocked in blocking solution (10% donkey serum, 0.1% PBS-Tween or 1% BSA, 0.1% PBS-Tween) for 1 h at RT and then incubated O/N at 4 °C with primary antibodies (Supplementary Table 6). Secondary anti-rabbit/mouse/goat/chicken antibodies coupled to Alexa Fluor®-647, 555, 488, and 750 fluorochrome conjugates (Life Technologies) were incubated for 90 min at 1:250 dilution in blocking solution. Sections were counterstained with DAPI 1 μg/ml (ThermoFisher, 62248) for 10 min, washed, and mounted with prolonged diamond antifade reagent (ThermoFisher, P36970). Immunofluorescence images were captured using an LSM 980 confocal microscope (Zeiss) and a slide scanner (3Fhistech Panoramic 250 Flash II). Image analysis and quantification were performed with ImageJ software (v.2.14.0/1.54f). Quantitative collagen and EYFP expression analysis was digitally performed with Visiopharm software (Horsholm, Denmark). The presence and staining intensity of stromal collagen was detected and labeled by automated threshold classification, using three classes of collagen: absent; loose; intense. The absolute and relative amount of EYFP-positive glandular epithelial cells were detected and labeled by automated threshold classification, using two classes: EYFP negative vs. EYFP positive.

### Immunohistochemistry (IHC)

FFPE sections (4 μm) were deparaffinized and rehydrated using xylene and ethanol. H&E and IHC, (Supplementary Table 6) were performed. Stainings for CK8, VIMENTIN, and αSMA were performed by the Translational Research Unit (TRU) at the Institute of Tissue Medicine and Pathology, University of Bern or performed in-house for marker p63 (Supplementary Table 6). For IHC, antigens were retrieved by incubating sections in antigen unmasking solution (citrate-based buffer, Adipogen, H-3300) for 20 min. Sections were blocked for 10 min in 30% H_2_O_2_, followed by 30 min RT incubation in 1% BSA in PBS-0.1% Tween 20. Normal mouse (Adipogen, I-2000-1) IgGs were used as control. Anti-mouse EnVision+System-HRP (DAKO, K400111-2) was used to develop the primary antibody, followed by counterstaining with hematoxylin and mounting with Aquatex. Image acquisition was performed with a slide scanner (3Dhistech Panoramic 250 Flash II).

### Digital histopathological evaluation

Scanned murine prostate tissue sections stained with H&E and different IHC markers were provided as indicated in (Supplementary Table 4.1). Images were captured using a Pannoramic 250 Flash II scanning microscope (3Dhistech). H&E slides were assessed for any relevant histopathologic changes by performing blinded histopathological grading by an independent pathologist (S.d.B). Quantitative analysis was performed to measure the extent of neoplastic growth and level of invasion. In a descriptive and semi-quantitative approach, the following features were assessed: Inflammatory stromal response; epithelial-mesenchymal transition; stromal desmoplasia; and cystic distention of mPIN.

The area of neoplasia and invasive growth was assessed on CK8 stained sections with Viopharm software (2022.11, Horsholm, Denmark) in the following workflow: (1) Prostate tissue was manually defined as a region of interest (ROI), separately for benign and neoplastic tissue; (2) Detection and labeling of CK8 positive epithelium and glandular lumen (automated, deep learning classification); (3) Manual label corrections were performed where needed; (4) Epithelial label was manually relabeled as ‘invasive’ where present; (5) The following outputs were measured and calculated: *total area (mm*^*2*^*)* and *percentage of* (i) *benign*, (ii) *neoplastic* and (iii) *invasive prostate epithelium; total count of invasive proliferation*.

### Biophysical organoids characterization—flow-based method

The W8 system (CellDynamics iSRL) is a standardized technique that detects the terminal velocity tracked by the sample’s motion when free-falling into a dedicated flow channel while the flow is at rest [43]. Analyses were performed on fixed organoids cultured for 1 week, then exposed to PFA 4% at room temperature for 1 h, washed twice with phosphate-buffered saline (PBS), and stored in PFA 0.04% for up to 1 month. Before measurements, organoids were moved into a 15 ml tube and resuspended in 7 ml W8 Analysis Solution (WAS, CellDynamics) at low concentration (<200 organoids/ml). 20–40 organoids for every tested condition were selected and analyzed in terms of mass density (fg/μm^3^), diameter (μm), and weight (ng) according to the protocol established by Cristaldi et al. [43].

It is important to underline that mass density accounts for the totality of the biological constituents within the analyzed organoid, which differs from the commonly adopted terms of density often intended as a cell counting reference (i.e., cell density, seeding number). For such reason, when combined with precise size and weight measurements, it permits a crucial correlation between physical-derived data [43] and biological-correlated events in the 3D cell culture field. Such biophysical characterization has been recently associated with several aspects ranging from extracellular matrix growth kinetics [44] to drug activity effect [39], well as to viability and permeation [45] to maturation monitoring for regenerative medicine purposes [46]. Moreover, it allows for a key impact on truly perceiving the population’s heterogeneity, coupled with organoids selection and separation for enhanced quality control.

Data were carried out in three replicates per single organoid, followed outlier identification and exclusion from the dataset based on box and whiskers distributions. One-way ANOVA statistical tests were used, followed by Tukey’s post hoc test. *P*-values less than 0.05 were considered significant.

### Microvasculature-on-chip

The microfluidic device, chip fabrication process, cell seeding, chip maintenance, immunostaining, and permeability assay have been previously described in detail [30, 31]. Briefly, human umbilical vein endothelial cells (HUVEC, Gibco) and normal human lung fibroblasts (NHLF, Lonza) were suspended in 2 U/mL bovine plasma thrombin (Sigma) in endothelial basal medium 2 (EMB2, Lonza) at final concentration of 4 ×10^7^ and 1 ×10^7^ cells/mL, respectively. For cell seeding, HUVECs, NHLFs, and fibrinogen solution were mixed at a ratio of 1:1:2 and immediately seeded into the central chamber. After 10 min, EGM2 was loaded in microchannels, and the reservoirs were filled. All chips were incubated for 7 days. After 6 days of coculturing, some of the chips were treated with conditioned media from NPK, NPKC, and NPK^OECripto^ cells. The remaining were used for loading NPK, NPKC, and NPK^OECripto^ cells inside the microvasculature network and live imaging for 24 h in CQ1 Confocal Imaging Cytometer Yokogawa with the respective analysis software CellPathFinder (Cenibra GMBH). All chips treated with separate cell populations, or their conditioned media, were incubated for 24 h. After live imaging, the chips containing cancer cells were utilized for immunostaining targeting VE-CADHERIN, F-ACTIN, and DAPI.

Extravasation efficiency represents the number of cells that have extravasated from the microvasculature towards hydrogel. The quantification was performed on Z-projected confocal images in which the three channels (EYFP+ cells (green), VE-CADERIN (red), and DAPI (blue)) were overlayed.

### Western blotting

The protein extraction from both tissue and matched organoids was performed using RIPA buffer with Protease and Phosphatase Inhibitor Cocktail (PPC1010-1ML, Sigma Merck).

The protein concentrations were quantified using Qubit^TM^ Protein and Protein Broad Range (BR) Assay Kits (Q33221, Thermofisher). 30 μg from each sample were immunoblotted using precast-gel (4-20% Mini-PROTEAN® TGX^TM^ Precast Protein Gels, Biorad). Immunoblots were incubated with the following antibodies: CRIPTO1 (Abcam, ab19917), β-ACTIN (Cell Signaling, 4967), PTEN (Cell Signaling, 9559S), pAKT (Ser473) (Cell Signaling, 9271S), AKT (Cell Signaling, 4691-S), NKX3.1 (Cell Signaling, 83700T), EGFP (Abcam, ab13970), K-RASG12D (GeneTex, GTX635362)(Supplementary Table 6). The membranes were incubated with the same 1:1000 primary antibody concentration (except for CRIPTO-1 1:300) and with 1:2500 secondary antibody (ECL Mouse IgG, HRP-linked whole Ab from mouse, NA931VS and ECL Rabbit IgG, HRP-linked whole Ab from donkey NA934VS, GE Healthcare; Rb anti-goat, HRP, Dako, P0449). The signals were detected using the FUSION FX7 Imaging System.

### Bulk RNA barcoding and sequencing (BRB-seq)

Transcriptomic analysis of bulk murine prostate tissues and organoids was performed. RNA was extracted from snap-frozen organoids by homogenization in Tripure Isolation Reagent (Sigma). Only RNA samples having between >200 ng and 1000 ng and with an RNA integrity number >6 were used. Samples were sent to Alithea Genomics SA (Lausanne, Switzerland) for library preparation and sequencing using extraction-free multiplexed 3’-end bulk RNA barcoding and sequencing (MERCURIUS^TM^ BRB-seq service [47]). The generation of bulk RNA Barcoding and sequencing (BRB-seq) libraries was performed using the MERCURIUS^TM^ DRUG-seq library preparation kit for Illumina and following the manufacturer’s manual (Alithea Genomics, #10841). All libraries were sequenced on Illumina Novaseq 6000.

Read trimming, alignment, and quantification steps were performed by Alithea Genomics using STARsolo v2.7.9a [47, 48] and the GRCm38.103 (mouse) reference genome. Utilizing the parameters -soloUMIdedup NoDedup 1MM_Directional”, and -quantMode GeneCounts” [49], we generated raw and UMI-deduplicated count matrices, opting for non-deduplicated counts for subsequent analyses.

RNA-seq raw counts were analyzed using DESeq2 (version 1.34.0) [50] keeping the genes which have at least 3 UMI counts for at least 3 samples. Differentially expressed genes were defined by applying an adjusted *p-*value threshold of 0.1. Functional enrichment analysis was performed using Enrichr (version June 8, 2023) [51] and mSigDB Hallmarks (version 2020) [52], BioPlanet (version 2019) [53] and Gene Ontology (version 2023) [54] databases. MYC targets signature scoring was calculated using Gene Set Variation Analysis (GSVA; version 1.42.0) [55].

### Statistical analysis

Statistical analyses were performed using Unpaired *t*-test, two-sided Mann-Whitney *U*-test, one-way ANOVA, and two-way ANOVA with multiple comparison testing as indicated in each figure legend. Unless specified, GraphPad Prism 10 software (GraphPad Software Inc.) or R-studio version 2023.06.1, build 524 were used for all statistical analyses and data visualization applied to the experimental data.

Data are presented as mean ± SD of at least three independent experiments unless stated in the figure legend. Statistical significance is presented as *p ≤ 0.05, **p ≤ 0.01, ***p ≤ 0.001, **** *p* ≤ 0.0001.

## Supplementary information

Supplementary Information

Supplementary Table S2

## Data Availability

The transcriptomics data are deposited to the Gene Expression Omnibus (GEO) with accession number GSE262362.
