# Supplementary material for: CRIPTO’s multifaceted role in driving aggressive prostate cancer unveiled by in vivo, organoid, and patient data
Source: Oncogene. 2024 Nov 26;44(7):462–75. doi: 10.1038/s41388-024-03230-x (PMC11810784; doi:10.1038/s41388-024-03230-x)
Supplement: Supplementary file 1 — Supplementary Information [file 41388_2024_3230_MOESM1_ESM.docx]

Supplementary Information

**CRIPTO’s multifaceted role in driving aggressive prostate cancer unveiled by in vivo, organoid, and patient data.**

Rodrigues Sousa et al.

^1^Urology Research Laboratory, Department for BioMedical Research, University of Bern, Bern, Switzerland

^2^COMPATH, Institute of Animal Pathology, University of Bern, Bern, Switzerland

^3^Department of Urology, Inselspital, Bern University Hospital, University of Bern, Bern, Switzerland

^4^Organs-on-chip Technologies Laboratory, ARTORG Center for Biomedical Engineering Research, University of Bern, Bern, Switzerland

^5^Department for BioMedical Research, Translational Organoid Research, University of Bern, Bern, Switzerland

^6^Huntsman Cancer Institute, Department of Oncological Sciences, University of Utah, Salt Lake City, USA

^7^CellDynamics i.s.r.l., Bologna, Italy

^8^Department of Cardiology, Leiden University Medical Center, Leiden, The Netherlands

^9^Department of Cell and Chemical Biology, Leiden University Medical Center, Leiden, The Netherlands

**Summary of Supplementary Figures**

Figure S1.1: Description of a tissue microarray of primary prostate tissue part of the EMPaCT.

Figure S1.2: Gating strategy to sort dissociated and labeled cells from fresh mouse prostate tissue with flow cytometry.

Figure S1.3: Characterization of prostate tumor growth in 6-month-old experimental mice.

Figure S2: Supplementary histopathological characterization of mouse prostate cancer phenotypes.

Figure S3.1: Mouse prostate cancer organoids are amenable to genetic manipulation.

Figure S3.2: Representative brightfield and immunofluorescence images of whole mount stainings prepared from organoids.

Figure S3.3: Cell viability analysis using CellTiter-Glo 3D luminescent cell viability assay.

Figure S3.4: Microfluidic chip specification, live imaging, and conditioned media experiment.

Figure S3.5: Effects of Conditioned Media overexpressing CRIPTO on organoids’ morphology.

Figure S4: Supplementary data for morphological characterization of mouse prostate organoids.

Figure S5: Supplementary transcriptomics results and MYC signature.

**Summary of Supplementary Tables**

Table S1: List of genotypes of the genetically engineered mouse models (GEMMs) used in this study.

Table S2: Summary of clinical and survival data

Table S3: List of oligonucleotides used in this study.

Table S4.1: Labeling and available IHC staining of the examined prostate tissue sections in *Castration Setting.*

Table S4.2: Summary of histopathologic phenotypic analyses of mouse prostate tumors in *Castration Setting.*

Table S4.3 Immunofluorescence (IF) analyses of mouse prostate tumor stroma in *Castration Setting*.

Table S5: Efficiency formation of organoids

Table S6: List of antibodies for immunofluorescence (IF), immunohistochemistry (IHC), and Western Blot.

Supplementary Information (Supplementary Figures)

**CRIPTO’s multifaceted role in driving aggressive prostate cancer unveiled by in vivo, organoid, and patient data.**

Rodrigues Sousa et al.


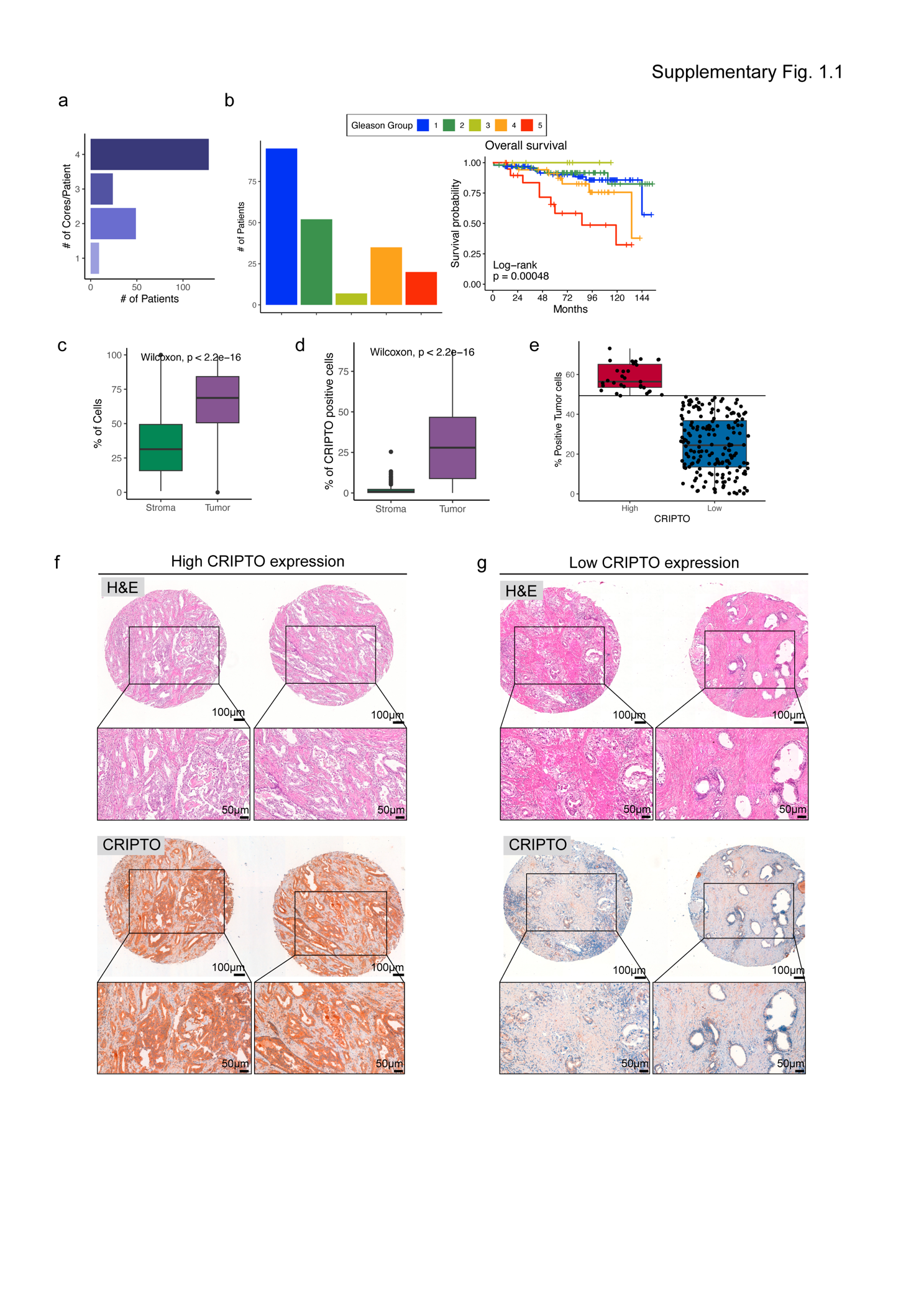


**Figure S1.1:** **Description of a tissue microarray of primary prostate tissue part of the EMPaCT. a** Representation of tumor samples derived from the index lesion, which includes more differentiated cores of each tumor (from 1 to 4) per sample. **b** Curve analysis of overall survival based on Gleason score clusterization (Gleason groups 1, 2, 3, 4, 5) (log-rank, **** *p-*value = 0.00048). **c** Percentage of stroma and tumor (epithelium) cells (Wilcoxon signed-rank test, **** *p-*value < 0.001). **d** Percentage of CRIPTO positive cells (%) in stromal and epithelial tissues (Wilcoxon signed-rank test, *****p-*value < 0.0001). **e** Representation of the % of high- and low- CRIPTO-positive tumor cells. **f, g** H&E (haematoxylin and eosin) staining (top) and validation of the protein expression of CRIPTO by immunohistochemistry (bottom) in representative cases of (**f**) high-CRIPTO expression group and (**g**) low-CRIPTO expression group. Scale bar = 100 μm and 50 μm (higher magnification).

**
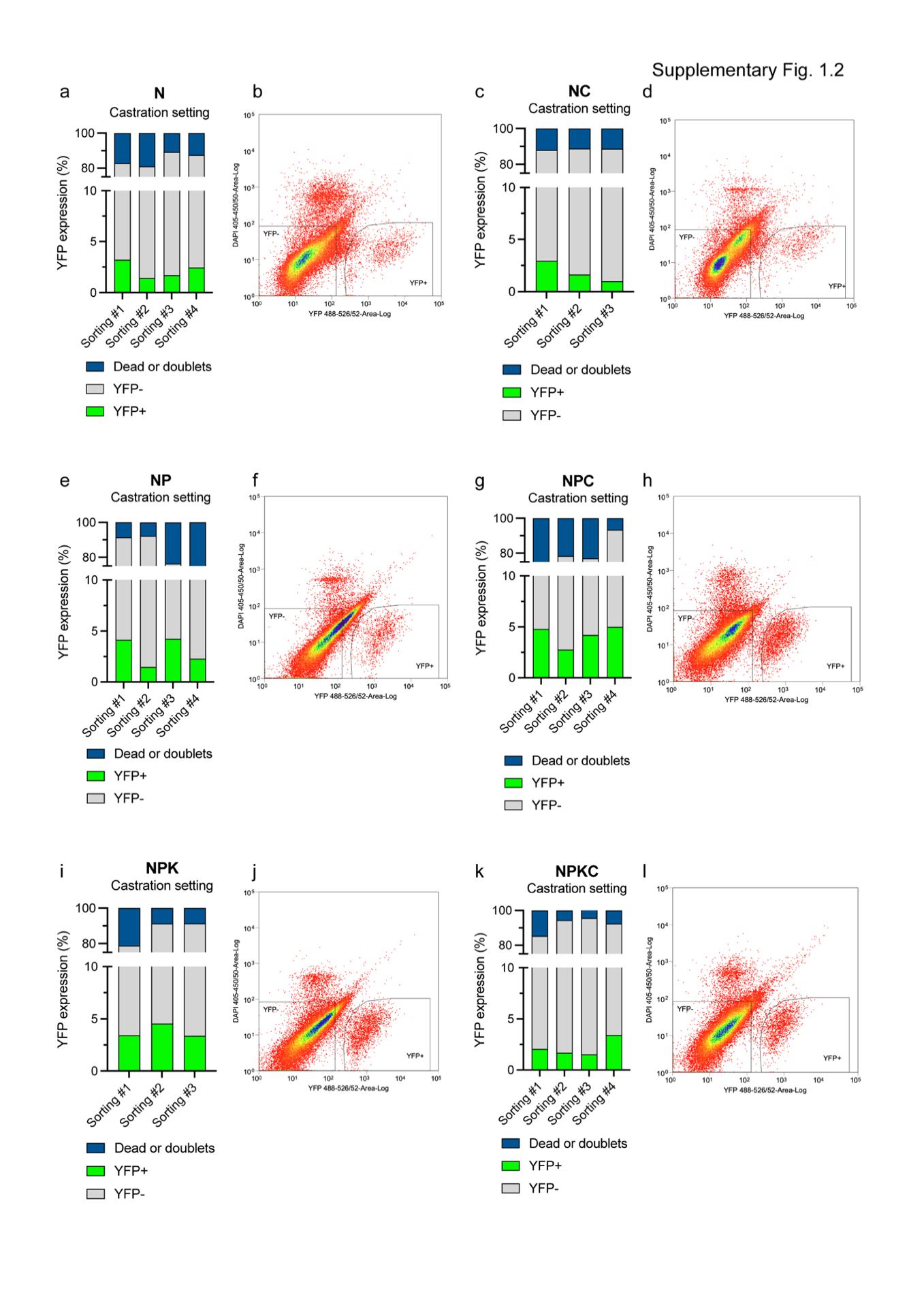
**

**Figure S1.2: Gating strategy to sort dissociated and labeled cells from fresh mouse prostate tissue with flow cytometry.** Flow cytometry sorting of EYFP-positive mouse prostate cells. **a, c, e, g, i, k** Fluorescence-activated cell sorting (FACS) of EYFP-marked cells derived from mouse prostate tissue in different experimental FACS sorting replicates (n_N_=4; n_NC_=3; n_NP_=4; n_NPC_=4; n_NPK_=3; n_NPKC_=4). **b, d, f, h, j, l** Final scatter plot of cell sorting gating strategy determined by fluorescence of EYFP. Briefly, the cells are first gated based on the forward scatter (FSC-A) and side scatter (SSC-A (log)) properties. Negative cells have the same scatter properties as EYFP-positive cells. Then, the single-cell population is selected based on FSC-H and FSC-W. The dead cell population is excluded based on the intensity of DAPI fluorescence detected on the 405-450 channel. Subsequently, the EYFP-positive cells are identified considering the EYFP fluorescence collected on the 488-562/52 channel. The sorting was done with a Beckman Coulter MoFlo ASTRIOS cell sorter using Summit v6.3 software.


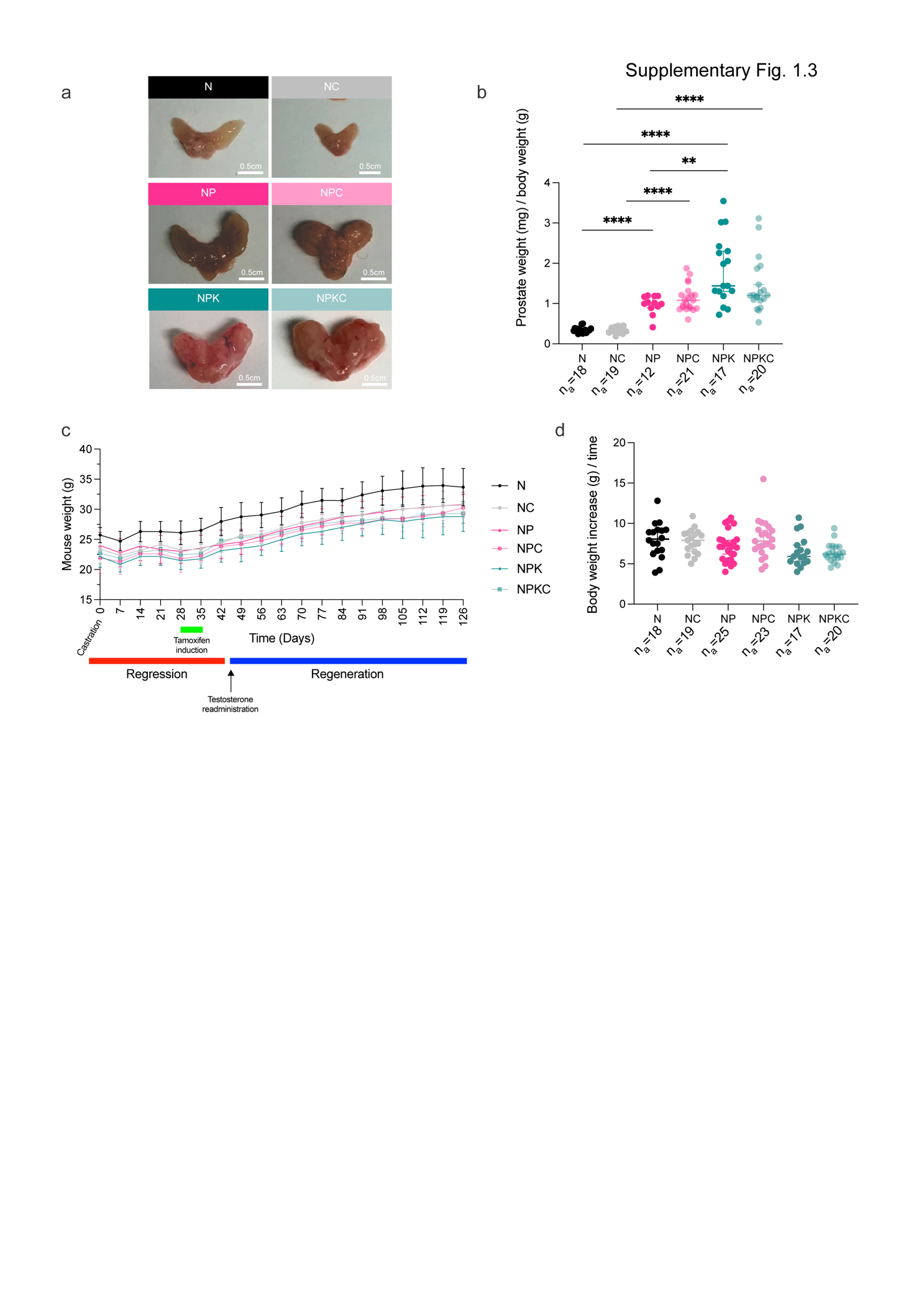


**Figure S1.3: Characterization of prostate tumor growth in 6-month-old experimental mice. a** Representative macroscopic pictures of mouse prostate from different genotypes (N, NC, NP, NPC, NPK, and NPKC). Scale bar = 0.5 cm. **b** Dot plots show relative prostate weight (n_N_=18, n_NC_=19, n_NP_=12, n_NPC_=21, n_NPK_=17, n_NPKC_=20). Mean weights (mg) of the whole prostate were normalized to total body weight (g) at the experimental endpoint (two-tailed unpaired *t-*test, N vs. NP, **** *p-*value < 0.0001; N vs. NPK, **** *p-*value < 0.0001; NP vs. NPK, ** *p-*value = 0.002; NC vs NPC, **** *p-*value < 0.0001; NC vs NPKC, **** *p-*value < 0.0001; N vs. NC, *p-*value = 0.93; NP vs. NPC, *p-*value = 0.16; NPK vs. NPKC, *p-*value = 0.10). The center lines show the mean, and the error bars depict SD. **c** Male average weekly body weight (g) from 8 to 24 weeks of age in different genotypes (N, NC, NP, NPC, NPK, and NPKC) (two-tailed unpaired *t*-test, N vs. NC, ** *p-*value = 0.004; NP vs. NPC, *p-*value = 0.27, NPK vs. NPKC; *p-*value = 0.31). **d** Male mice average overall gain (g) between the start and experimental end-point (two-tailed unpaired *t-*test, mean values for N = 7.927g (n=18); NC = 7.763g (n=19); NP = 7.188 (n=25); NPC = 8.056 (n=23); NPK = 6.535 (n=17); NPKC = 6.355 (n=20), *p-*value > 0.05).

**
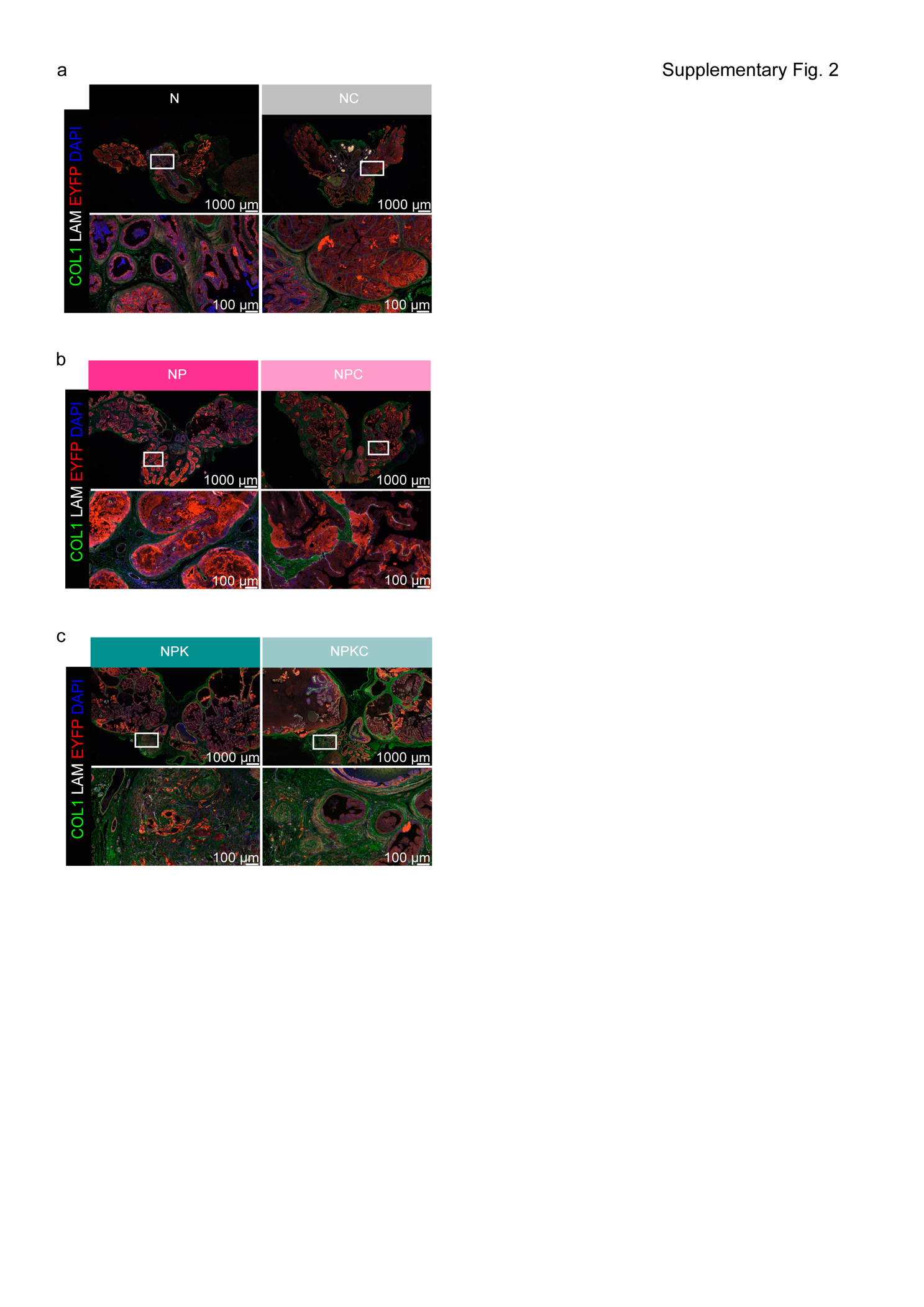
**

**Figure S2: Supplementary histopathological characterization of mouse prostate cancer phenotypes.** **a-c** Representative IF images for stromal markers: collagen I, COL1 (green), laminin, LAM (white), EYFP (red), and nuclear stain, DAPI (blue) in N background (**a**), NP background (**b**), and NPK background (**c**). Scale bar = 1000 μm and 100 μm (higher magnification). All images represent the *Castration Setting* (previously described).

**
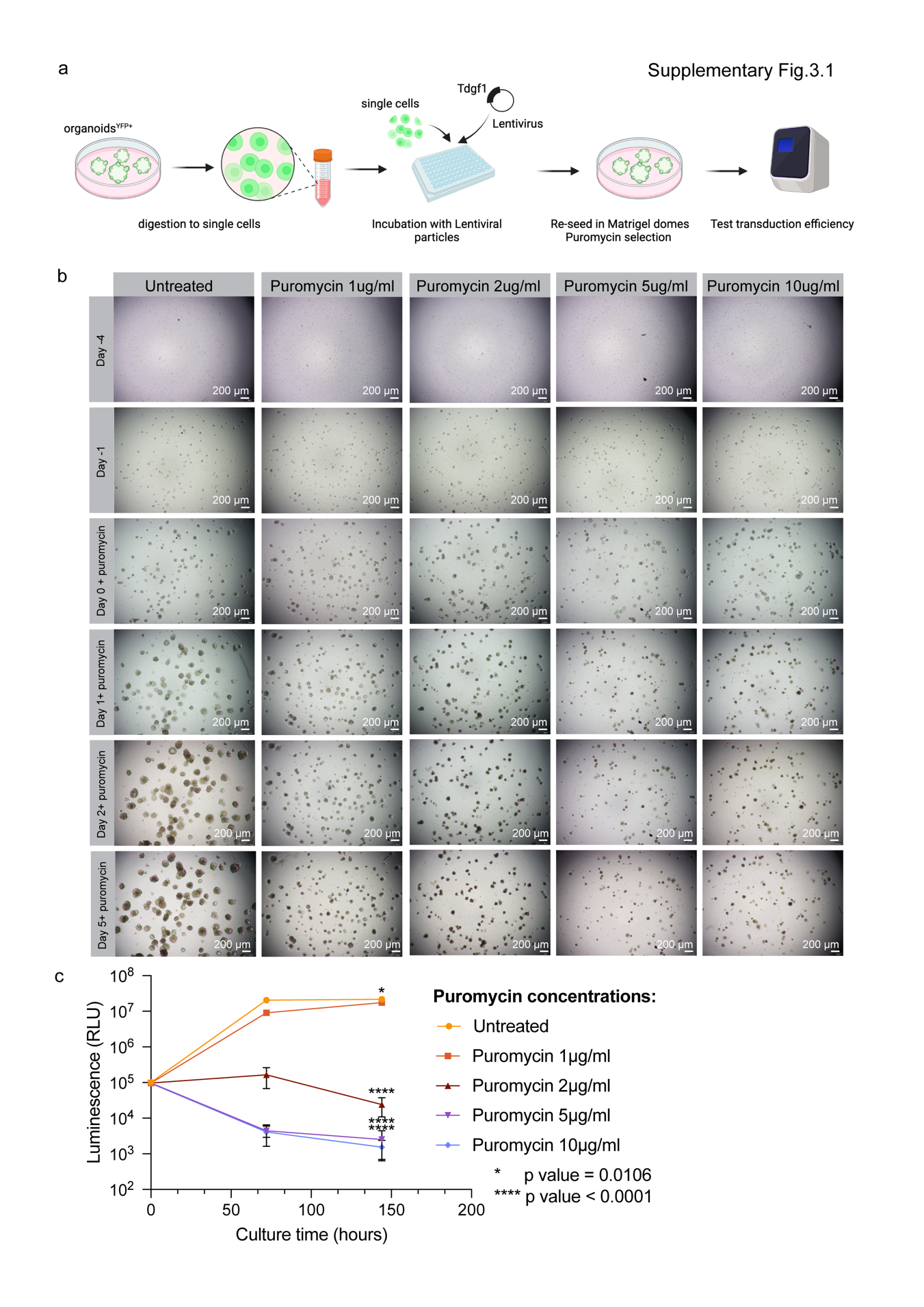
**

**Figure S3.1: Mouse prostate cancer organoids are amenable to genetic manipulation.** Lentiviral transduction *in vitro* permits the overexpression of *Cripto* on organoids. **a** Schematic overview of viral transduction of murine prostate cancer organoids. Created with Biorender.com. **b** Brightfield images of representative NPC^EYFP+^ organoids used for puromycin concentration selection from 4 days (day -4) before puromycin selection and 5 days post puromycin (day +5) addition. Scale bar = 200 μm. **c** Growth curve comparing different puromycin concentrations. The *p-*value shown for 120h was estimated by Tukey’s multiple comparisons against the untreated group (* *p-*value = 0.0106, **** *p-*value = 0.0001).

**
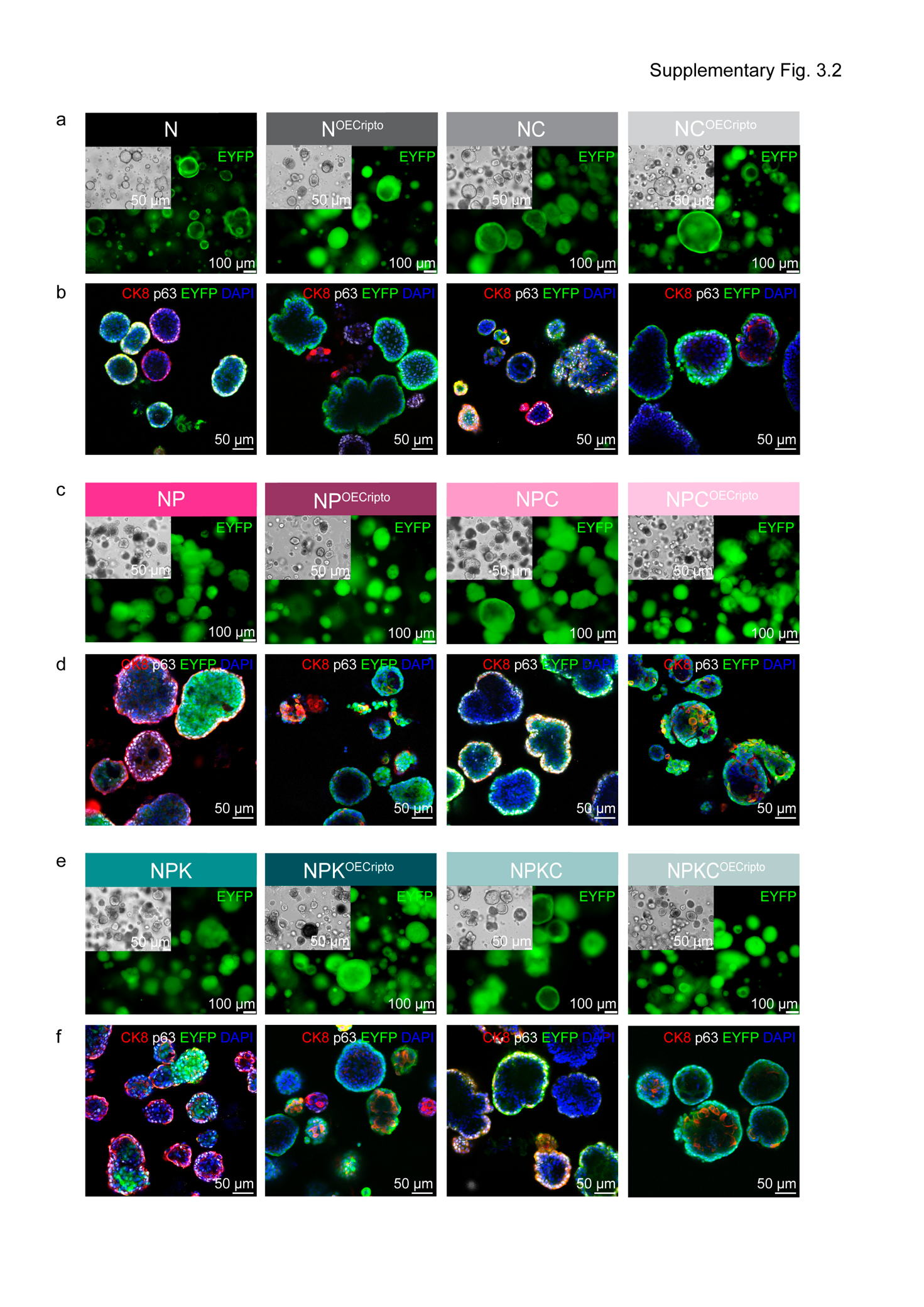
**

**Figure S3.2: Representative brightfield and immunofluorescence images of whole mount stainings prepared from organoids.** Mouse prostate organoids replicate prostate tissue histology. **a, c, e** Representative brightfield and fluorescent (EYFP+) images of organoids for different genotypes (N, N^OECripto^, NC, NC^OECripto^, NP, NP^OECripto^, NPC, NPC^OECripto^, NPK, NPK^OECripto^, NPKC, NPKC^OECripto^). Scale bar = 50 μm (brightfield) and 100 μm (IF). **b, d, f** Representative IF images of staining for CK8 (red), p63 (white), EYFP (green) and nuclear stain, DAPI (blue). Scale bar = 50 μm.

**
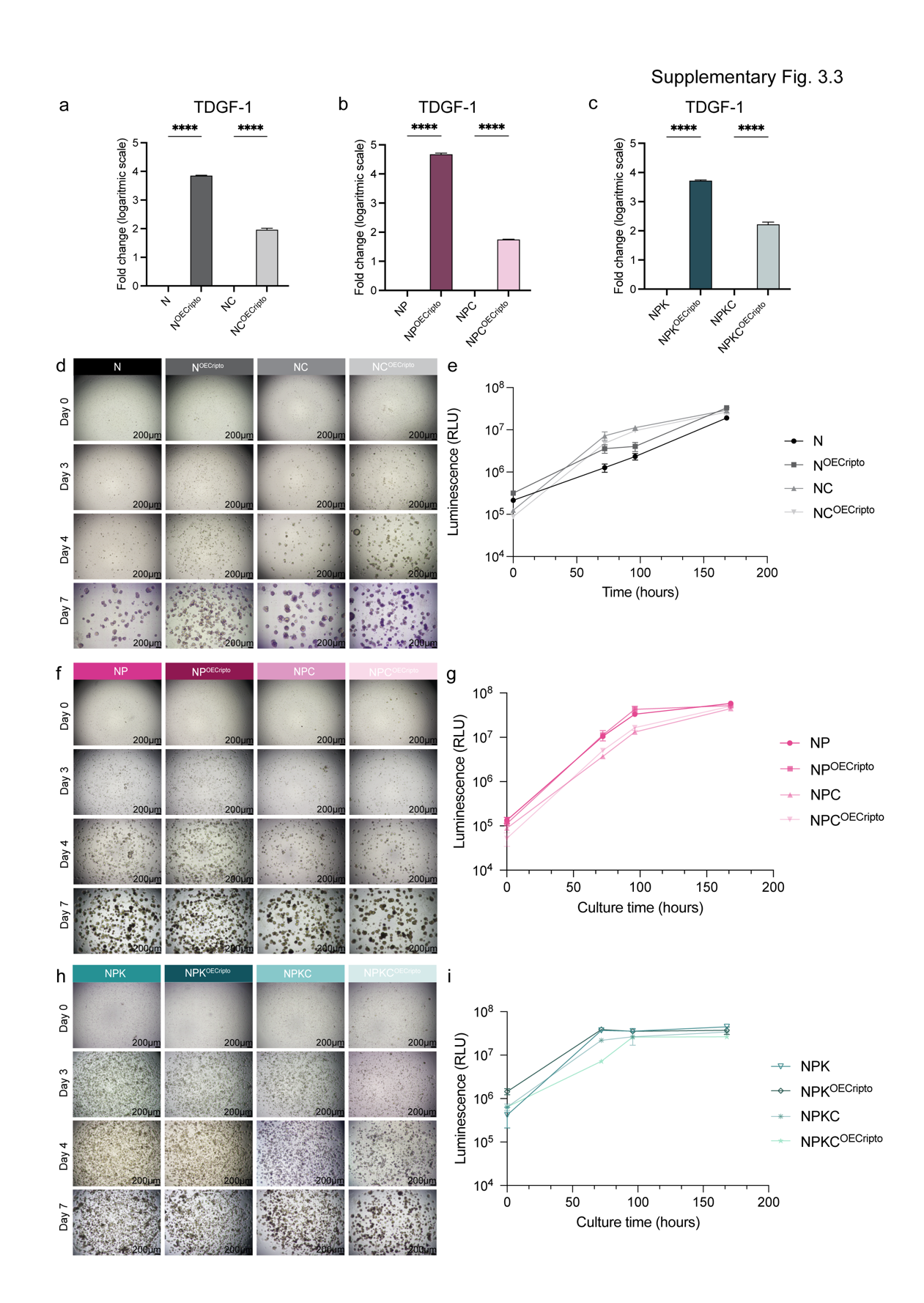
**

**Figure S3.3: Cell viability analysis using CellTiter-Glo 3D luminescent cell viability assay.** Molecular characterization of transduced organoids and combined analyses of cell viability. **a-c** Real-time PCR analyses of TDGF1 gene expression in transduced organoids with expression levels of mRNA indicated as fold change normalized to relative control in (**a**) N background, (**b**) NP background, and (**c**) NPK background (two-tailed unpaired *t-*test, ***** p*-value < 0.0001). **d, f, h** Representative brightfield pictures of organoid culture over time. Scale bar = 200 µm. **e, g, i** CellTiter-Glo® 3D Cell Viability assays of EYFP cells from organoids cultures. The activity index is defined as a ratio of luminescence values (n=5) at 72, 96, and 168 hours relative to day 0 of seeding in all three different backgrounds (N (**e**), NP (**g**), and NPK (**i**)) including CRIPTO knockout (NC, NPC, NPKC) and CRIPTO overexpressed groups (N^OECripto^, NP^OECripto^, NPK^OECripto^, NC^OECripto^, NPC^OECripto^, NPKC^OECripto^).

**
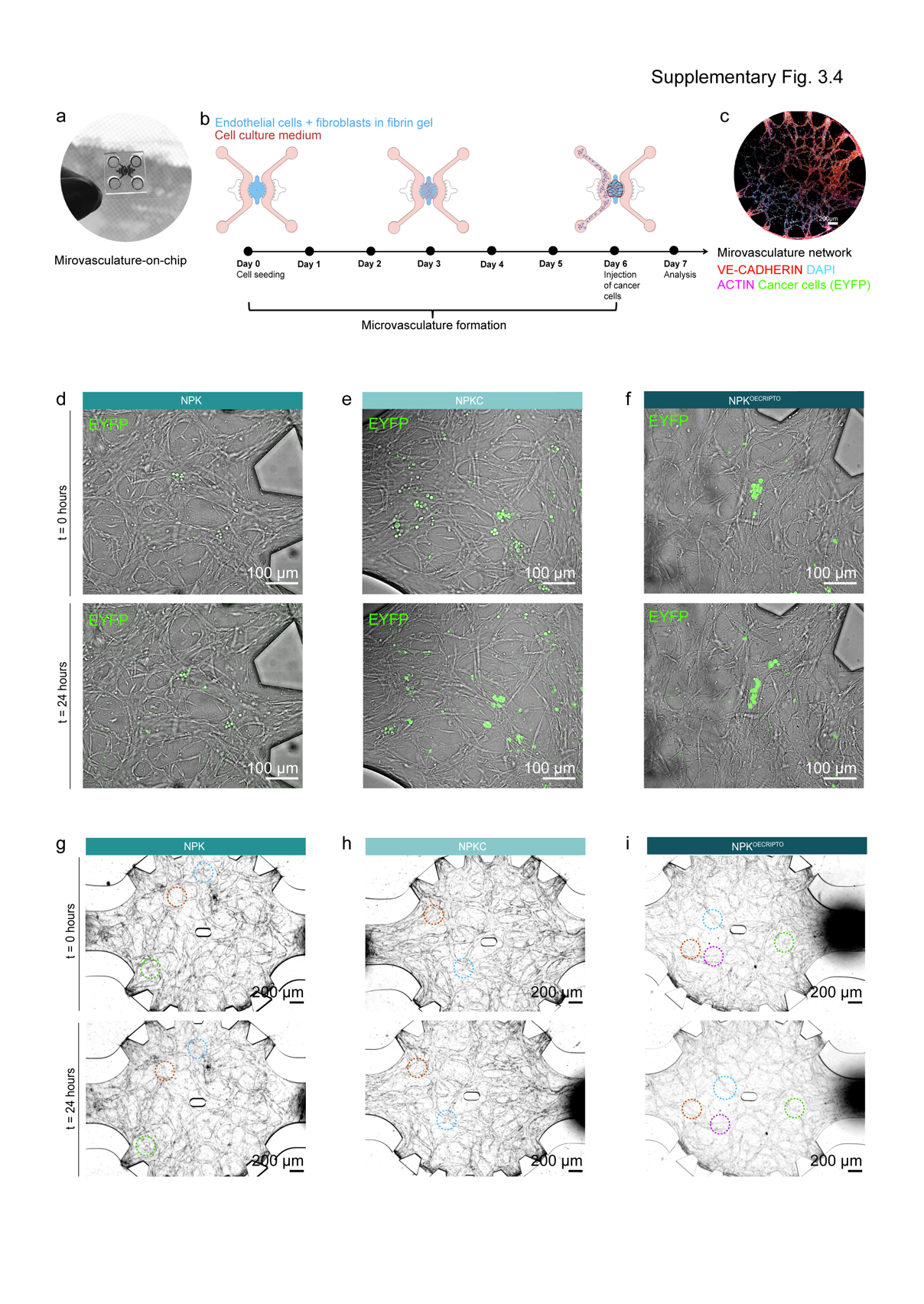
**

**Figure S3.4: Microfluidic chip specification, live imaging, and conditioned media experiment. a** Microfluidic chip fabricated by PDMS soft lithography and bonded to coverslip. **b** Schematic overview of chip seeding process (seven days). Coculture of endothelial cells (HUVECs) and fibroblasts are resuspended in fibrin gel and loaded in the central chamber (blue); in the two outermost chambers (green), fibroblasts resuspended in fibrin gel are loaded; culture media (red) is added in the two symmetric microchannels. **c** Representative IF staining showing the microvasculature network through the expression of VE-CADHERIN (red), the cancer cells through the expression of EYFP (green), actin filaments through the expression of ACTIN (magenta), and nuclear stain, DAPI. Scale bar = 200 µm. **d-f** Representative images of live imaging (experimental t=0 and t = 24h) in the three groups conditions NPK (**d**), NPKC (**e**), and NPK^OECripto^ (n_chips_=4 per group condition) (**f**). Images represent a segment of the central chamber and are overlays of the green channel (EYFP) and bright field. Scale bar = 200 µm. **g-i** Representative bright-field images of central chamber microvasculature network in NPK (**g**), NPKC (**h**), and NPK^OECripto^ (**i**) groups at t = 0 and t = 24h. The Microvasculature network was treated with conditioned media (organoids mouse medium). Circle regions indicate microvessel rupture after culture with conditioned media. Scale bar = 200 µm.

**
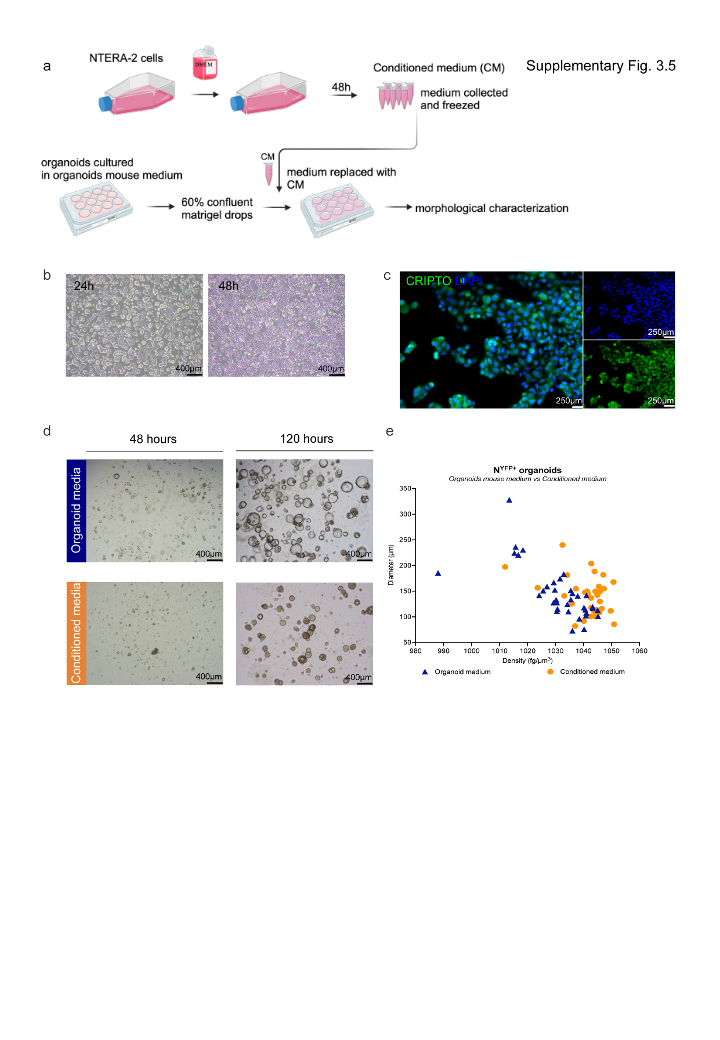
**

**Figure S3.5: Effects of Conditioned Media overexpressing CRIPTO on organoids’ morphology. a** Schematic overview of culture conditions applied to N organoids culture. Created with BioRender.com **b** Representative brightfield images of NTERA cells at 24 and 48 hours. Scale bar = 100 μm. **c** Representative IF staining of CRIPTO and nuclear stainind DAPI. Scale bar = 250 μm **d** Organoids cultured with organoid media (top) or conditioned media (bottom) at 48 and 120 hours. **e** Representative case of multiple variables plot of mass density (fg/µm^3^) and diameter (µm) of N^YFP+^ organoids cultured with Organoid medium (blue) or Conditioned medium (orange).

**
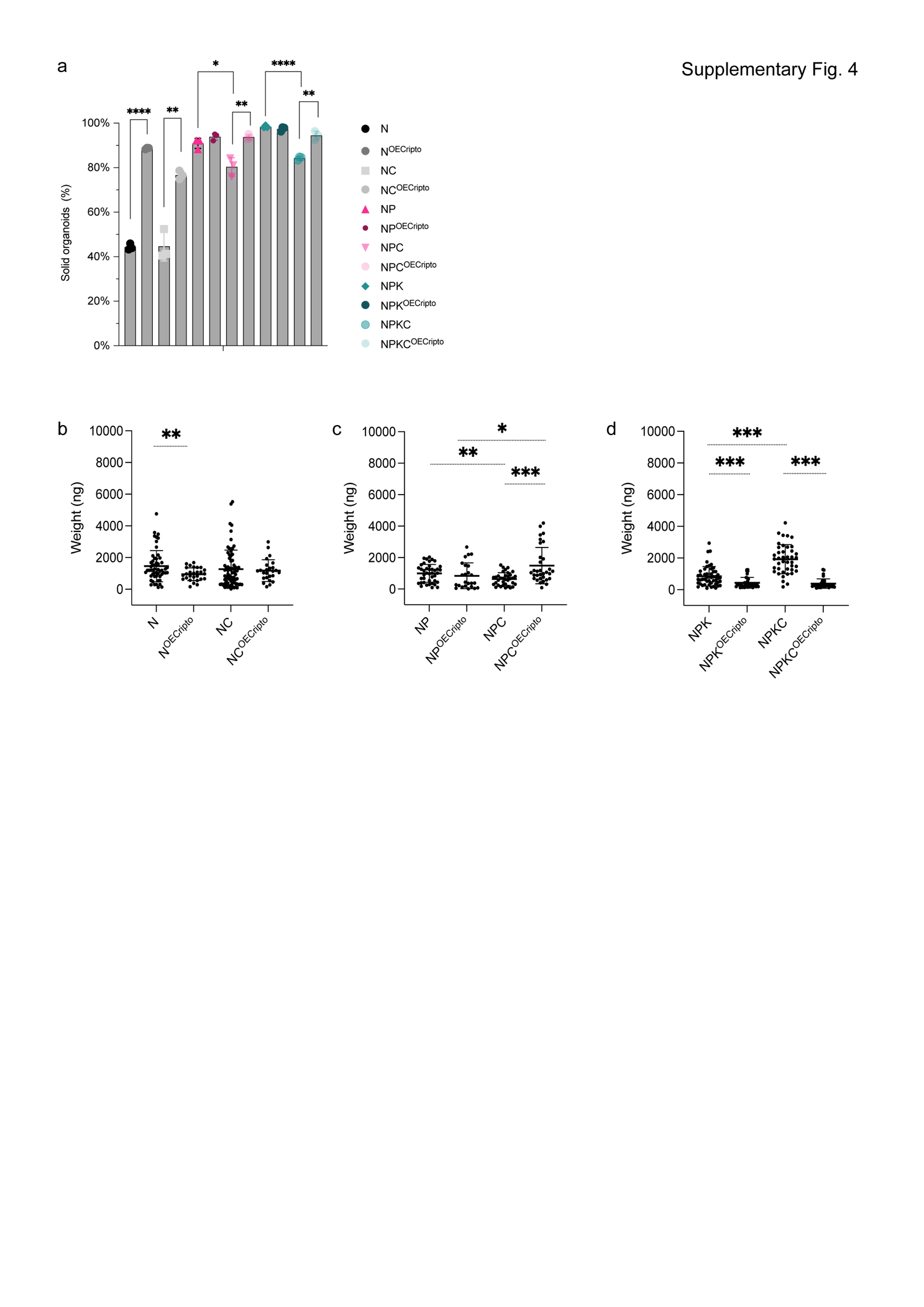
**

**Figure S4: Supplementary data for morphological characterization of mouse prostate organoids.** **a** % of solid mouse prostate organoids morphology over the total analyzed Matrigel domes (n_domes_=3) per genotype (two-tailed, unpaired *t-*test, N vs. N^OECripto^, **** *p-*value < 0.0001; NC vs. NC^OECripto^, ** *p-*value = 0.0013, NP vs. NPC, * *p-*value 0.017; NPC vs NPC^OECripto^, ** *p-*value = 0.005; NPK vs. NPKC, **** *p*-value <0.0001; NPKC vs. NPKC^OECripto^, ** *p-*value = 0.0013). **b-d** Physical paramter (weight) assessed with W8 system (ng) (two-sample *t-*test, * *p*-value <0.05, ** *p*-value < 0.01, *** *p*-value < 0.001).

**
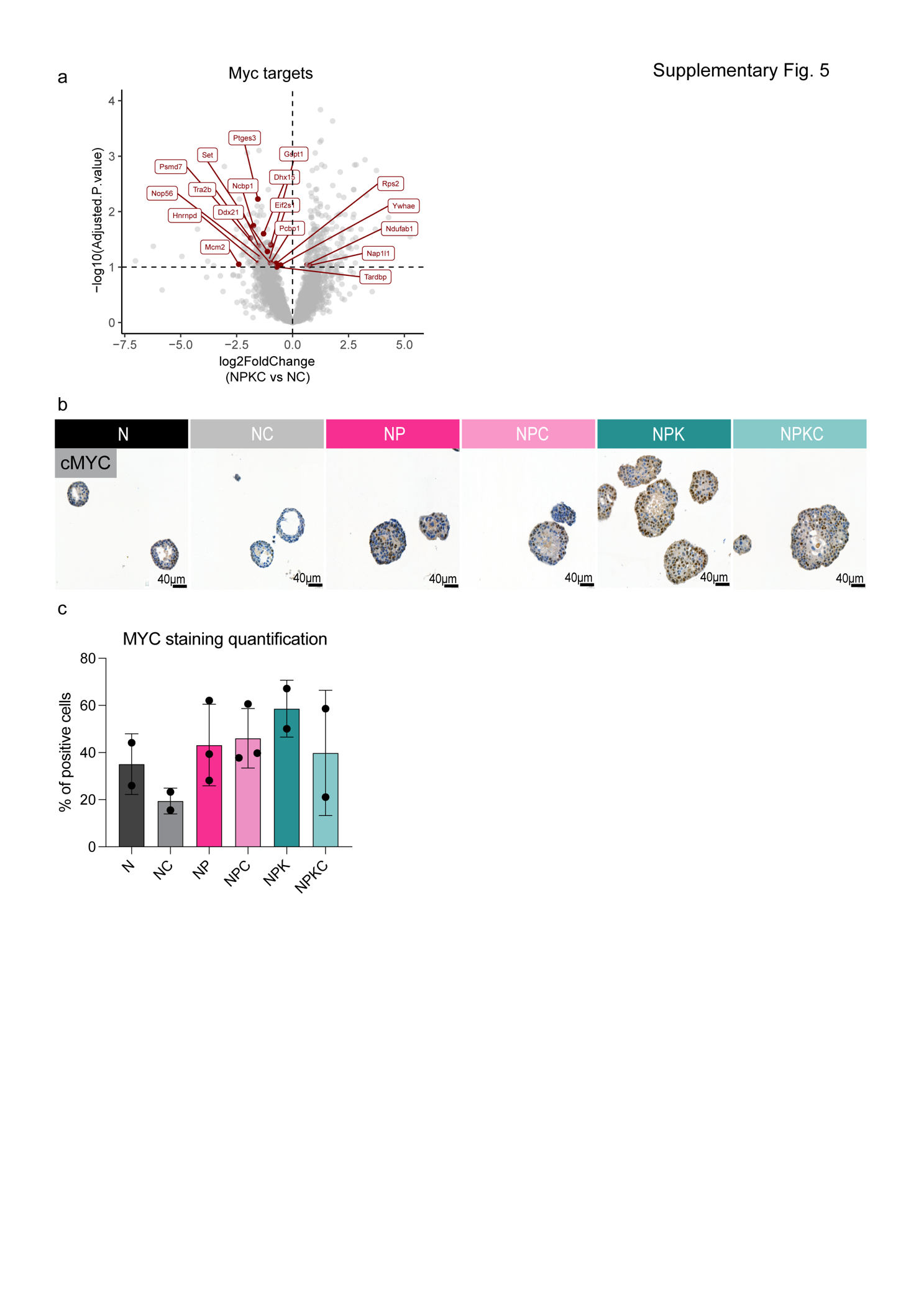
**

**Figure S5:** **Supplementary** **transcriptomics results and MYC signature. a** Volcano plot of NPKC vs NC differential expression analysis results, highlighting the identified MYC targets. **b** Representative validation of the protein expression of c-MYC by immunohistochemistry in all organoid groups (N, NC, NP, NPC, NPKC). Scale bar = 40 μm. **c** Quantitative analyses of total % of positive c-MYC epithelial cells. Mean values of positive cells per genotypes conditions are the following: N = 35.08%, NC = 19.43%, NP = 43.19 %, NPC = 46.06 % NPK = 58.64%, NPKC = 39.85% (Two-tailed Mann-Whitney test, N vs. NC, *p-*value = 0.33; NP vs. NPC, *p-*value > 0.99; NPK vs. NPKC, *p-*value = 0.66; N vs. NP, *p*-value = 0.80; N vs NPK, *p-*value = 0.33; NP vs NPK, *p-*value = 0.40).

Supplementary Information (Supplementary Tables)

**CRIPTO’s multifaceted role in driving aggressive prostate cancer unveiled by in vivo, organoid, and patient data.**

Rodrigues Sousa et al.

| Acronym | Genotype |
| --- | --- |
| N | *Nkx3.1*^CreERT2/CreERT2^; *R26R*^EYFP/EYFP^ |
| NP | *Nkx3.1*^CreERT2/CreERT2^; *Pten*^flox/flox^; *R26R*^EYFP/EYFP^ |
| NPK | *Nkx3.1*^CreERT2/CreERT2^; *Pten*^flox/flox^; *K-ras*^LSL/+^; *R26R*^EYFP/EYFP^ |
| NC | *Nkx3.1*^CreERT2/CreERT2^; *Cripto*^flox/flox^; *R26R*^EYFP/EYFP^ |
| NPC | *Nkx3.1*^CreERT2/CreERT2^; *Pten*^flox/flox^; *Cripto*^flox/flox^; *R26R*^EYFP/EYFP^ |
| NPKC | *Nkx3.1*^CreERT2/CreERT2^; *Pten*^flox/flox^; *K-ras*^LSL/+^; *Cripto*^flox/flox^; *R26R*^EYFP/EYFP^ |

**Table S1. List of genotypes of the genetically engineered mouse models (GEMMs) used in this study.**

**Table S2: Summary of clinical and survival data.**

| Purpose and name | Sequence | |
| --- | --- | --- |
| Genotyping Allele | Forward primer 5' - 3' | Reverse primer 5' - 3' |
| *Nkx3.1*^CreERT2/CreERT2^ | CTCCGCTACCCTAAGCATCC | GACACTGTCATATTACTTGGACC |
| *Pten*^flox/flox^ | CAAGCACTCTGCGAACTGAG | AAGTTTTTGAAGGCAAGATGC |
| *K-ras*^LSL/+^ | TGT CTT TCC CCA GCA CAG T | GCA GGT CGA GGG ACC TAA TA |
|  | CTG CAT AGT ACG CTA TAC CCT |  |
| *R26R*^EYFP/EYFP^ | CTG GCT TCT GAG GAC CG | CAG GAC AAC GCC CAC ACA |
|  | AGG GCG AGG AGC TGT TCA | TGA AGT CGA TGC CCT TCA G |
| *Cripto*^flox/flox^ | TGG TGA TCC AGA GTC ATT GG | GGG GTC ATT CCT CTC CTA GC |
| Real Time qPCR | Forward primer 5' - 3' | Reverse primer 5' - 3' |
| *Tdgf-1* | GATGTTCGCAAAGAGCACTG | CTGAGGAAGACAGTGGAGCTG |
| *Vim* | CGGCTGCGAGAGAAATTGC | CCACTTTCCGTTCAAGGTCAAG |
| *Tgfβ-1* | CTCCCGTGGCTTCTAGTGC | GCCTTAGTTTGGACAGGATCTG |
| *Krt8* | TCCATCAGGGTGACTCAGAAA | CCAGCTTCAAGGGGCTCAA |
| *Krt5* | GTCAGGACTGAGGAGAGGGA | TGTCCAGGACCTTGTTCTGC |
| *Actb* | CACTGTCGAGTCGCGTCC | TCATCCATGGCGAACTGGTG |
| *Hprt* | GTTGGGCTTACCTCACTGCT | TCATCGCTAATCACGACGCT |

**Table S3: List of oligonucleotides used in this study.**

| Group | Replicate | H&E | CK8 | p63 | VIM | αSMA | cMYC |
| --- | --- | --- | --- | --- | --- | --- | --- |
| N | 1 | x | x | x | x | x | x |
|  | 2 | x |  |  |  |  | x |
|  | 3 | x |  | x |  |  | x |
|  | 4 | x |  |  |  |  |  |
|  | 5 | x |  |  |  |  |  |
| NC | 1 | x | x | x | x | x | x |
|  | 2 | x |  |  |  |  | x |
|  | 3 | x |  |  |  |  | x |
|  | 4 | x |  | x |  |  |  |
| NP | 1 | x | x | x | x | x | x |
|  | 2 | x | x | x |  |  | x |
|  | 3 | x | x | x |  |  | x |
| NPC | 1 | x | x | x | x | x | x |
|  | 2 | x | x | x |  |  | x |
|  | 3 | x |  |  |  |  | x |
|  | 4 | x |  |  |  |  |  |
|  | 5 | x |  | x |  |  |  |
|  | 6 | x | x |  |  |  |  |
| NPK | 1 | x | x | x | x | x | x |
|  | 2 | x | x | x |  |  | x |
|  | 3 | x | x | x |  |  | x |
|  | 4 | x | x | x |  |  | x |
|  | 5 | x | x | x |  |  |  |
|  | 6 | x | x |  | x | x |  |
| NPKC | 1 | x | x | x | x | x | x |
|  | 2 | x | x | x |  |  | x |
|  | 3 | x | x | x |  |  | x |
|  | 4 | x | x | x |  |  |  |
|  | 5 | x | x | x |  |  |  |
|  | 6 | x |  |  |  |  |  |
|  | 7 | x | x | x | x | x |  |

**Table S4.1: Labeling and available IHC staining of the examined prostate tissue sections in *Castration Setting.*** The number of replicates for each genotype is indicated in the rows, while the stainings performed are shown in the columns. H&E, haematoxylin and eosin. VIM, Vimentin. αSMA, alpha-smooth muscle actin.

|  |  | Normal | | PIN | | mPIN + microinvasion | | mPIN + microinvasion + invasive carcinoma | |
| --- | --- | --- | --- | --- | --- | --- | --- | --- | --- |
| Group | N | n_of samples_ | % | n_of samples_ | % | n_of samples_ | % | n_of samples_ | % |
| N | 5 | 5 | 100 | 0 |  | 0 |  | 0 |  |
| NC | 4 | 4 | 100 | 0 |  | 0 |  | 0 |  |
| NP | 3 | 0 |  | 1 | 33.3 | 2 | 66.7 | 0 |  |
| NPC | 6 | 0 |  | 5 | 83.3 | 1 | 16.7 | 0 |  |
| NPK | 6 | 0 |  | 0 |  | 1 | 16.7 | 5 | 83.3 |
| NPKC | 7 | 0 |  | 0 |  | 3 | 42.9 | 4 | 57.1 |

**Table S4.2: Summary of histopathologic phenotypic analyses of mouse prostate tumors in *Castration Setting.*** Analysis at the end of the experiment (after castration, prostate regression, induction, prostate regeneration, and testosterone re-administration). Tamoxifen induction was performed at 3 months of age, after 1 month from castration (performed at 2 months of age). N, the total number of prostate tissues analyzed per group (N, NC, NP, NPC, NPK, NPKC). Phenotype, pathological description of the histological phenotype according to the classification of Park et al. 2022.

|  | Collagen type I | | | | | | |
| --- | --- | --- | --- | --- | --- | --- | --- |
| Group | Assessed stroma area (mm^2^) | Area collagen intense (mm^2^) | Area collagen loose (mm^2^) | Total collagen | Area collagen intense (%) | Area collagen loose (%) | Total collagen (%) |
| N | 5.2 | 0.5 | 1.0 | 1.5 | 9.8 | 18.9 | 28.7 |
| N | 9.3 | 0.6 | 2.1 | 2.6 | 6.0 | 22.4 | 28.5 |
| N | 6.1 | 0.2 | 1.2 | 1.4 | 3.4 | 18.9 | 22.3 |
| NC | 9.1 | 0.9 | 2.0 | 2.9 | 9.9 | 21.4 | 31.3 |
| NC | 12.2 | 0.7 | 1.7 | 2.3 | 5.6 | 13.7 | 19.3 |
| NC | 10.0 | 0.8 | 1.4 | 2.1 | 7.6 | 13.9 | 21.5 |
| NP | 25.9 | 3.9 | 10.1 | 14.0 | 14.9 | 38.9 | 53.8 |
| NP | 20.9 | 2.7 | 9.2 | 11.9 | 12.8 | 44.0 | 56.8 |
| NP | 20.8 | 2.7 | 8.1 | 10.7 | 12.8 | 38.7 | 51.5 |
| NPC | 17.0 | 1.5 | 6.4 | 7.9 | 8.8 | 37.6 | 46.3 |
| NPC | 18.6 | 1.4 | 6.1 | 7.5 | 7.7 | 32.7 | 40.4 |
| NPC | 16.7 | 2.3 | 7.9 | 10.1 | 13.6 | 47.2 | 60.8 |
| NPK | 23.0 | 1.8 | 8.0 | 9.9 | 8.0 | 34.8 | 42.8 |
| NPK | 21.0 | 2.5 | 7.4 | 9.9 | 11.8 | 35.1 | 46.9 |
| NPK | 20.0 | 4.3 | 8.3 | 12.6 | 21.4 | 41.7 | 63.1 |
| NPKC | 25.7 | 4.4 | 9.1 | 13.5 | 17.0 | 35.5 | 52.5 |
| NPKC | 20.8 | 0.7 | 6.3 | 6.9 | 3.2 | 30.1 | 33.3 |
| NPKC | 32.5 | 1.0 | 8.2 | 9.2 | 3.2 | 25.1 | 28.3 |

**Table S4.3: Immunofluorescence (IF) analyses of mouse prostate tumor stroma in *Castration Setting*.** Quantitative analysis of stromal marker (COL1, collagen type I) in *Castration Setting*. The area of collagen expression was detected and measured digitally. Based on a defined IF staining intensity threshold, collagen was classified as either loose or intense. Values are indicated as absolute values (mm^2^) and percentages (%).

|  | Efficiency formation | | |
| --- | --- | --- | --- |
| Group | Drop1 (%) | Drop2 (%) | Drop3 (%) |
| N | 2.3143 | 2.3143 | 3.3571 |
| N^OECripto^ | 6.0714 | 6.5714 | 6.0571 |
| NC | 2.9429 | 4.2000 | 3.0000 |
| N^COECripto^ | 5.3571 | 4.6857 | 6.0714 |
| NP | 4.0429 | 3.1000 | 2.4429 |
| NP^OECripto^ | 4.5857 | 4.7000 | 4.7143 |
| NPC | 3.9571 | 3.7714 | 3.4429 |
| NPC^OECripto^ | 7.2857 | 6.9714 | 6.7857 |
| NPK | 4.3286 | 5.9857 | 7.5857 |
| NPK^OECripto^ | 7.6714 | 9.0286 | 6.2429 |
| NPKC | 3.1429 | 3.0429 | 3.4714 |
| NPKC^OECripto^ | 5.1571 | 7.6286 | 6.1571 |

**Table S5: Efficiency formation of organoids.** Efficiency formation (%) measurements in indicated genotypes measured by the total number of organoids formed per Matrigel® dome (n_dome_=3) over the total number of single cells seeded at day 0 of culture (7000 single cells).

| Antibody | Company | Catalog No | Clone | Use and dilution | | |
| --- | --- | --- | --- | --- | --- | --- |
|  |  |  |  | IF | IHC | Western Blot |
| Rabbit monoclonal Anti-AR | Abcam | ab133273 | EPR1535(2) | 1 to 100 | 1 to 100 |  |
| Rabbit polyclonal Anti-αSMA | Abcam | ab5694 |  |  | 1 to 200 |  |
| Mouse polyclonal Anti-αSMA | Sigma | A2547 |  | 1:20000 |  |  |
| Rabbit monoclonal Anti-Ck8 | Abcam | ab53280 | EP1628Y | 1 to 200 | 1 to 400 |  |
| Rabbit monoclonal Anti-Cripto | Abcam | ab19917 |  |  |  | 1 to 300 |
| Rabbit monoclonal Anti-Cripto | Produced by Spike *et al.*^1^ |  | PBL6900 |  | 1 to 1000 |  |
| Rabbit monoclonal anti-cMYC | Cell Marque | 395R-15 | EP121 |  | 1 to 50 |  |
| Goat polyclonal Anti-Collagen I | Southern Biotech | 1310-01 |  | 1 to 200 |  |  |
| Goat polyclonal Anti-GFP DyLight 488 | Bio-Techne | NBP1-69969 |  | 1 to 200 |  |  |
| Chicken polyclonal Anti-GFP | Abcam | ab13970 |  | 1 to 500 |  |  |
| K-rasG12D | GeneTex | GTX635362 |  |  |  | 1 to 1000 |
| Rabbit polyclonal Anti-Laminin | DAKO | Z0097 |  | 1 to 500 |  |  |
| Mouse monoclonal Anti-p63 | Santa Cruz | sc-25268 |  | 1 to 500 |  |  |
| Pten | Cell Signaling | 9559S |  |  |  | 1 to 1000 |
| Rabbit monoclonal Anti-Vimentin | Abcam | ab92547 | EPR3776 |  | 1 to 2000 |  |
| Goat IgG, control antibody | Vector laboratories | I-5000-5 |  | 1 to 200 |  |  |
| Mouse IgG, control antibody | Vector laboratories | I-2000-1 |  | 1 to 500 |  |  |
| Rabbit IgG, control antibody | Vector laboratories | I-1000-5 |  | 1 to 200 |  |  |
| Goat anti-mouse IgG, Alexa Fluor 647 | ThermoFisher | A21241 |  | 1 to 250 |  |  |
| Donkey anti-rabbit IgG, Alexa Fluor 555 | ThermoFisher | 62248 |  | 1 to 250 |  |  |

**Table S6: List of antibodies for immunofluorescence (IF), immunohistochemistry (IHC), and Western Blot.**

Supplementary Methods

**CRIPTO’s multifaceted role in driving aggressive prostate cancer unveiled by in vivo, organoid, and patient data.**

Rodrigues Sousa et al.

*Organoid-forming efficiency assays*

Organoid forming efficiency was assessed by quantification of the organoid number formed per dome (n=3). Briefly, 8-bit binary images of the whole organoid dome were analyzed with ImageJ2 (v.2.9.0) using the “cell counter” option set. Then, the number of organoids formed per dome was normalized to the total number of single cells seeded on day 0 of the culture.

*Organoids’ morphological characterization and viability*

Brightfield pictures of organoid cultures were analyzed at passages 3-4 (p3-4) after 7 days of culture. Organoid morphologies were defined as solid or hollow and manually determined on brightfield images of 5 Matrigel domes (technical replicates) per genotype condition using the cell counter in ImageJ2 (v.2.9.0).

For viability assay, single cells from dissociated organoids (Dispase II for 1h and TrypLE Express for 20 min at 37°C) at p5-8 (depending on infection protocol) were seeded at a cell density of 6,000 cells per 7 μl Matrigel dome (n=5 domes) in a 96-well plate (Sarstedt, 833.924) and cultured in 100ul of Mouse organoid medium for 7 days. Viability was assessed with CellTiter-Glo® 3D kit (Promega, G9683) at 0, 72, 96, and 168 hours according to the manufacturer’s instructions. Briefly, 100ul of CellTiter-Glo® 3D reagent was added to each well followed by 5 min of shaking of plates and 25 min incubation at 37°C. After incubation, volumes were transferred into 96-well half-area plates (Costar, 3693), and luminescence was measured using Varioskan Lux Microplate Reader (ThermoFisher).

*Mouse prostate tissue fixation and preparation for immunostaining*

Entire mouse prostates were harvested, washed in PBS, and fixed in 4% PFA (Sigma, P6148) and paraffin-embedded using standard protocols. Formalin-fixed paraffin-embedded (FFPE) blocks were sectioned (4 μm-thick sections), collected onto glass slides, and dried O/N at 37°C.

*Organoid fixation and preparation for immunostaining*

Organoids cultured for 7 days in Matrigel® domes were washed with PBS, and extracellular matrix (ECM) was enzymatically digested with Dispase II for one hour. Samples were collected, centrifuged (150g, 5 min, 4°C), washed with PBS, and fixed with 4% PFA for 1h at RT. After washing with PBS, samples were ready for whole-mount staining (stored in PBS 0.4% PFA at 4°C for one month). At the same time, for sections-immunostaining, the organoids pellet was resuspended into 80 μl HistoGel (Histocom, 83009-992) domes for 1h and then placed in 50% ethanol and stored at 4°C O/N. HistoGel domes were then subjected to paraffin embedding.

*Whole-mount immunofluorescence staining of organoids*

Fixed-organoid suspension was collected in a 96-well Round (U) Bottom plate (Sigma, 92097) and spin down (150g, 5 min). PBS was removed, and organoids were permeabilized in 0.3% Triton-X for 10 min at room temperature, followed by blocking in 10% donkey serum in PBS/0.1% PBS-Tween for 1h at room temperature, gently rocking. Incubation with primary antibodies (Table S7) resuspended in blocking solution was performed O/N at 4°C gently rocking. After that, three times washing steps (150g, 5 min) in PBS/0.1% Tween were performed, followed by incubation in secondary anti-rabbit/mouse/goat antibodies coupled to Alexa Fluor®-647, 555, 488 fluorochrome conjugates (dilution 1:250, Life Technologies) and DAPI (dilution 1:1000) for 90 min in blocking solution at room temperature gently rocking. Organoids were washed gently rocking and transferred to flat glass bottom, black-walled 96-well plates (Corning, 4580) in PBS/0.1% PBS-Tween.

Confocal microscopes (LSM 710 with Airyscan or LSM980 with Airyscan, ZEISS) were used to image immunofluorescence staining.

*Cell culture and conditioned medium preparation*

NTERA-2 cells (obtained from ATCC) were cultured in T75 flasks with DMEM medium (ThermoFisher, 31966047) supplemented with 10% fetal calf serum (FCS; Sigma Aldrich, F7524), 1% penicillin-streptomycin (ThermoFisher, 15140122), and 1% GlutaMAX (ThermoFisher, 35050061) at 37°C in a humidified incubator with 5% CO_2_. The medium was replaced every 2-3 days, and cells were passaged at approximately 70-80% confluency. To prepare the conditioned medium, NTERA-2 cells were grown until 80% confluency, washed with PBS, and incubated for 48 hours at 37°Co with fresh serum-free DMEM medium. After 48 hours, the conditioned medium was collected, centrifuged at 1000g for 10 minutes, and filtered using a 0.2 μm filter (VWR, 156608). The conditioned medium was stored at -20°C until further use. N^EYFP+^ organoids were cultured in a conditioned medium after supplementing the organoids medium with NTERA-2 conditioned medium at a 1:1 ratio. The control organoids were cultured in organoid medium alone without any NTERA-2 conditioned medium. Organoids were collected, fixed, and morphological characterization with W8 technology was assessed as previously described.

*RNA extraction*

RNA was extracted from primary tissue and organoids using TRI Reagent (Sigma, 11667165001). Samples were homogenized in 500 μl TRI reagent and incubated for 5 minutes at room temperature. 100 μl of Chloroform was added and centrifuged at 10,000x*g* for 10 minutes. The aqueous phase was transferred to a new 1.5 Eppendorf tube, and 500 μl of isopropanol were added and incubated for 10 minutes at room temperature. Samples were centrifuged for 10 min at 10,000x*g*, and the pellet was consecutively washed with 100% and 75% ethanol. The pellet was air-dried for 15 minutes and resuspended in a maximum volume of 30 μl of RNAse-free water. Samples were stored at -20°C until further processing.

*Real-time quantitative PCR*Quantitative PCR was performed using FastStart Universal SBR Green Master (Rox) (Sigma, 4913914001) following the manufacturer’s instructions on QuantStudio3 applied biosystems (ThermoFisher Scientific). 1 μg of RNA was then reverse transcribed into cDNA with a Reverse Transcription kit (Promega) according to the manufacturer’s instruction using oligo (dT) primers (Promega) in a final reaction volume of 20 μL. qPCR was performed in 96- and 384-well format in a 10 μL reaction volume, including 2 ng of cDNA and gene-specific primers (Supplementary Table 3) using FastStart Universal SBR Green Master (Sigma, 4913914001). Following standard protocols, experiments were performed in three technical replicates. Data quality was assessed in the ThermoFisher ConnectTM Dashboard (ThermoFisher), and mean Cq values were extracted to Microsoft Excel for further analyses. ΔCq values were calculated and normalized to the loading controls *β-Actin* or *Hprt*. Fold-change gene expression was calculated with the normalized ΔΔCq values of N, NP, and NPK set to 1 (*n* = 3).
